# Development of a *DUX4*-targeting antibody oligonucleotide conjugate as a therapy for FSHD

**DOI:** 10.1093/nar/gkag301

**Published:** 2026-04-17

**Authors:** Barbora Malecova, David Sala, Garineh M Melikian, Rachel Johns, Gulin Erdogan, Marc Hartmann, Maryam Jordan, Joel Danny Arias, Arvind Bhattacharya, Qingying Meng, Oliver Dansereau, Samuel W Beppler, Venkata R Doppalapudi, Hanhua Huang, William Michael Flanagan, Arthur A Levin

**Affiliations:** Avidity Biosciences, Inc., 3020 Callan Rd, San Diego, CA 92121, USA; Avidity Biosciences, Inc., 3020 Callan Rd, San Diego, CA 92121, USA; Avidity Biosciences, Inc., 3020 Callan Rd, San Diego, CA 92121, USA; Avidity Biosciences, Inc., 3020 Callan Rd, San Diego, CA 92121, USA; Avidity Biosciences, Inc., 3020 Callan Rd, San Diego, CA 92121, USA; Avidity Biosciences, Inc., 3020 Callan Rd, San Diego, CA 92121, USA; Avidity Biosciences, Inc., 3020 Callan Rd, San Diego, CA 92121, USA; Avidity Biosciences, Inc., 3020 Callan Rd, San Diego, CA 92121, USA; Avidity Biosciences, Inc., 3020 Callan Rd, San Diego, CA 92121, USA; Avidity Biosciences, Inc., 3020 Callan Rd, San Diego, CA 92121, USA; Avidity Biosciences, Inc., 3020 Callan Rd, San Diego, CA 92121, USA; Avidity Biosciences, Inc., 3020 Callan Rd, San Diego, CA 92121, USA; Avidity Biosciences, Inc., 3020 Callan Rd, San Diego, CA 92121, USA; Avidity Biosciences, Inc., 3020 Callan Rd, San Diego, CA 92121, USA; Avidity Biosciences, Inc., 3020 Callan Rd, San Diego, CA 92121, USA; Avidity Biosciences, Inc., 3020 Callan Rd, San Diego, CA 92121, USA

## Abstract

Facioscapulohumeral muscular dystrophy (FSHD) is an autosomal dominant muscular disease in which genetic mutations activate *DUX4* expression in skeletal muscle. Currently, there are no approved therapies for FSHD. We developed *Delpacibart braxlosiran* (*del-brax*, also known as AOC 1020), an antibody oligonucleotide conjugate (AOC), for the treatment of FSHD that is designed to specifically target and reduce *DUX4* mRNA in skeletal muscle. AOC 1020 is composed of *DUX4* mRNA-targeting small interfering RNA (siRNA), siDUX4.6, conjugated to a human transferrin receptor 1 (TfR1)-targeting monoclonal antibody to facilitate productive siRNA delivery to muscle. We demonstrate that siDUX4.6 reduces DUX4-regulated gene expression in FSHD patient-derived myotubes *in vitro* and in skeletal muscle of the ACTA1-MCM; FLExDUX4 FSHD mouse model *in vivo*. Single systemic intravenous treatment was sufficient to prevent DUX4-induced muscle weakness and fibrosis in this FSHD mouse model and reduce DUX4-regulated genes by ∼75% 8 weeks post-dose. The pharmacokinetic profiles of AOCs with siDUX4.6 were comparable in murine and non-human primate muscle. These data demonstrate the potential of AOC 1020 to treat the underlying cause of FSHD by suppressing *DUX4* expression in muscles of patients with FSHD. The safety and efficacy of AOC 1020 is currently being investigated in clinical trials.

## Graphical Abstract

**Graphical Abstract:**
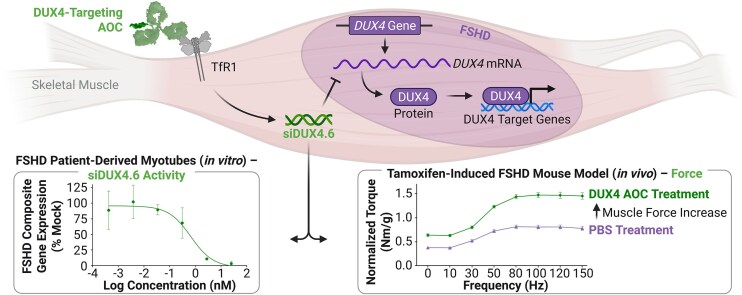

## Introduction

Facioscapulohumeral muscular dystrophy (FSHD) is a rare, debilitating, and progressive muscular dystrophy that affects 45 000–87 000 patients in the United States and Europe combined [1, 2]. Currently, there are no approved treatments for this disease [3]. Most patients experience symptom onset in adolescence or early adulthood; however, 7–15% have an earlier onset phenotype, frequently with a more severe and rapidly progressing disease [4]. The phenotype of skeletal muscle weakness and wasting is highly variable between patients and even within a patient, such that FSHD can and often does manifest asymmetrically. The disease is progressive, although at varying rates. While many different initial presentations have been described, almost any skeletal muscle can become affected, resulting in significant lifelong morbidity. Due to the variability in affected muscle groups, asymmetric distribution of muscle weakness, unpredictable rate of progression, and a general lack of disease awareness, the time from symptom onset to diagnosis frequently exceeds 10 years. Approximately 20% of patients become wheelchair dependent after 50 years of age [5–7]. Based on the significant clinical consequences of FSHD and the limited effectiveness of available treatment approaches, which are focused on symptom management, there remains a high unmet need for the development of disease-modifying therapies to treat the underlying cause of FSHD.

FSHD is classified into two types, FSHD1 and FSHD2, and an emerging consensus model of FSHD pathophysiology [8] is that both FSHD1 and FSHD2 are caused by genetic mutations leading to an ectopic expression of double homeobox 4 (*DUX4*) in skeletal muscle [9–12]. DUX4 is a transcription factor normally expressed during pre-implantation embryonic development (2- to 8-cell stage), after which it is epigenetically silenced in most adult tissues, including muscle, with the exception of testes and thymus, where its potential function is not yet well characterized [13–16]. Aberrant *DUX4* expression in skeletal muscle induces a network of DUX4-regulated genes, leading to a series of deleterious downstream events and muscle tissue degeneration characteristic of FSHD [8, 13, 16–19]. As *DUX4* is a transcription factor with low, sporadic, and highly variable expression in FSHD muscles [11, 20–22], measuring the expression levels of downstream genes induced by DUX4 is considered the most reliable way to assess DUX4 activity [16, 19, 23].

**Figure 1.**
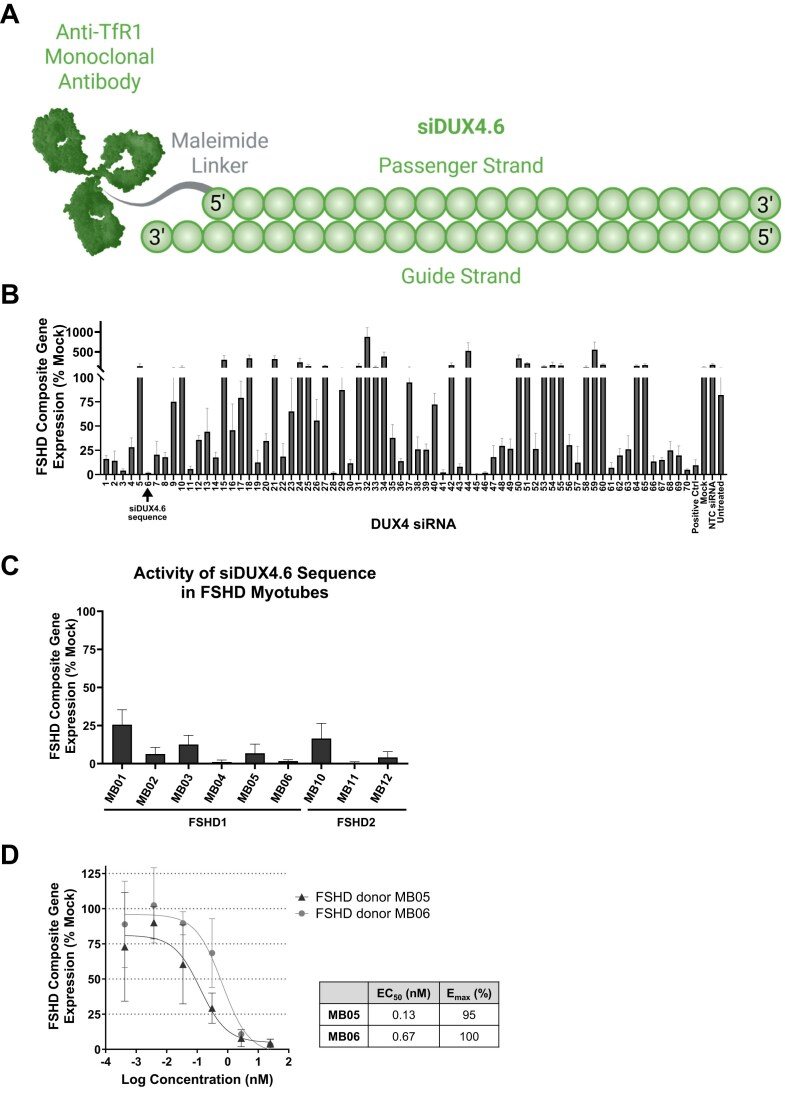
Illustration of AOC 1020 structure and activity evaluation of DUX4-targeted siRNAs in myotubes derived from FSHD patients. (**A**) Scheme of the AOC 1020 structure (drug-antibody ratio equal to 1). siDUX4.6 modification pattern is outlined in Supplementary Table S1. (**B**) Primary FSHD patient-derived myoblasts were transfected with *DUX4*-targeting siRNAs and induced to differentiate into myotubes to perform gene expression analysis. The FSHD composite score integrates the gene expression levels of four DUX4-regulated genes (*MBD3L2, ZSCAN4, LEUTX*, and *KHDC1L*). Screening of the *DUX4* siRNA library was performed in FSHD1 patient-derived primary myotubes MB06 at 10 nM final concentration. Data are represented as a percentage of mock transfection control (mean ± SD, *n* = 4 for library siRNAs, *n* = 8 for controls). (**C**) Activity of siDUX4.6 was evaluated across nine FSHD donor myotubes (MB06 data are the same as in panel B) (mean ± SD; *n* = 4 for siRNAs; *n* = 8 for mocks for MB03, MB04, and MB10; *n* = 4 for mocks for MB01, MB05, MB11, and MB12). Myotubes were collected 2 days (MB01, MB03), 3 days (MB02, MB05, MB07, MB10, MB11, MB12), or 4 days (MB04, MB06) after inducing differentiation (**D**). *In vitro* potency of the siDUX4.6 sequence was evaluated by concentration-response in two FSHD1 patient-derived primary myotubes (MB05, MB06). Data in the graphs are represented as a percentage of mock transfection control (mean ± SD, *n* = 4 for siRNA, *n* = 28 for mock). Log(inhibitor) versus response three-parameter calculation was used to fit the concentration-response curves. The best-fitting values for EC_50_ (half maximal effective concentration) and E_max_ (maximum response) are reported in the table. CTRL, control; FSHD, facioscapulohumeral muscular dystrophy; NTC, non-targeting control; siRNA, small interfering ribonucleic acid.

RNA-targeting approaches aimed at reducing *DUX4* expression in skeletal muscle are attractive therapeutic strategies to directly target the cause of FSHD [17]. Among these, a small interfering RNA (siRNA)-mediated therapeutic approach is highly attractive due to its sequence specificity for the target [24, 25]. Historically, the main challenge for the development of oligonucleotide-based therapies for muscle diseases has been the capability to efficiently deliver the oligonucleotides into skeletal muscle. We have developed an antibody oligonucleotide conjugate (AOC) platform that leverages the specificity of monoclonal antibodies (mAbs) for tissue delivery, including into skeletal muscle, together with the targeting precision of oligonucleotides [26]. We leveraged the AOC platform to develop AOC 1020 for the treatment of FSHD. AOC 1020 is an AOC composed of a monoclonal antibody (AV01mAb) targeting human transferrin receptor 1 (TfR1) that is conjugated to an siRNA oligonucleotide (siDUX4.6) targeting the *DUX4* mRNA (Fig. 1A). The AV01mAb component facilitates delivery of siDUX4.6 to muscle by binding to TfR1 on the cell surface and internalizing via receptor-mediated endocytosis [26]. The siDUX4.6 component provides the pharmacological mechanism of action, which is siRNA-mediated reduction of *DUX4* mRNA levels.

Here, we demonstrate that (i) siDUX4.6 reduces the expression levels of a DUX4-regulated gene signature in a variety of myotubes derived from individuals with FSHD, (ii) when siDUX4.6 is conjugated to a murine TfR1 mAb, it suppresses DUX4 activity and it has functional efficacy *in vivo* in a mouse model of FSHD, and (iii) when siDUX4.6 is conjugated to a human TfR1 mAb, the siRNA is distributed to skeletal muscle in non-human primates (NHPs). Taken together, this multi-pronged approach demonstrates the utility of AOC 1020 to modulate the course of FSHD. AOC 1020 is currently being evaluated in individuals with FSHD in the Phase 1/2 FORTITUDE™ (NCT05747924) and the Phase 2 FORTITUDE-OLE™ (NCT06547216) trials [27], and its activity supports advancing to the Phase 3 FORTITUDE-3 clinical trial (NCT07038200).

## Materials and methods

### *DUX4* siRNA library design and synthesis

*DUX4* transcript variant 1 sequence (NCBI Reference: NM_001306068.2) was used to design a library of 70 *DUX4*-targeting siRNAs. Sequences were selected leveraging *in silico* approaches to minimize off-target potential and predicted cell toxicity [28] (Supplementary Table S1).

### siRNA synthesis

siRNAs were synthetized with chemical modifications, including 2′-O-methyl (2′-OMe) and 2′-deoxy-2′-fluoro (2′-F) ribosugar modifications and phosphorothioate linkages for selected nucleotides, at Axolabs (Kulmbach, Germany).

### Antibody production

The human anti-TfR1 antibody was expressed in Chinese hamster ovary stable pools and purified using protein affinity chromatography, followed by hydrophobic interaction chromatography and anion exchange chromatography. The final antibody was buffer exchanged into either phosphate-buffered saline (PBS) or 50 mM sodium citrate buffer, pH 6.5, at a concentration of 20 mg/mL. Antibody purity was assessed by size exclusion chromatography.

### AOC generation

AOCs were generated using a standard random cysteine conjugation of siRNA and antibody using a maleimide linker [29]. The reaction mixture was purified using strong anion exchange chromatography to isolate the AOC with a drug-antibody ratio equal to 1. AOC fractions were concentrated, buffer-exchanged into PBS, and sterile filtered using a 0.2-μm filter. AOC purity was assessed using strong anion exchange chromatography, size exclusion chromatography, and sodium dodecyl-sulfate polyacrylamide gel electrophoresis (SDS-PAGE).

### Cell culture and transfection of primary myoblasts

FSHD patient-derived primary myoblasts were obtained from the University of Rochester (Supplementary Table S2). Myoblasts were grown in medium containing 80% Ham’s F-10 Nutrient Mix, 20% fetal bovine serum (FBS), 10 ng/mL recombinant human fibroblast growth factor, 1 µM dexamethasone, and penicillin and streptomycin. Myoblasts were seeded on Matrigel-coated 96-well plates (Costar) 24 h prior to transfection. The next day, *DUX4* siRNAs were formulated with Lipofectamine RNAiMAX^TM^ (Life Technologies, Waltham, MA, USA) and Opti-MEM^TM^ (Life Technologies, Waltham, MA, USA) according to the manufacturer’s “forward transfection” instructions. Myogenic differentiation was induced 24 h post-transfection with 15% KnockOut^TM^ Serum Replacement (KOSR)-containing differentiation medium as described previously [30].

### Cell collection and RNA isolation

Myotubes were collected in TRIzol and stored at −80°C until processing for RNA isolation using Direct-zol-96 RNA isolation kit (Zymo Research, Irvine, CA, USA) following the manufacturer’s protocol.

### Gene expression analysis in FSHD patient-derived myotubes

Purified RNA was converted to complementary DNA (cDNA) using the High-Capacity cDNA Reverse Transcription Kit (Applied Biosystems, Foster City, CA, USA). cDNA was analyzed by quantitative polymerase chain reaction (qPCR) using TaqMan Fast Universal Master Mix II (Thermo Fisher, Waltham, MA, USA), TaqMan probes for the genes of interest (Supplementary Table S3) (Thermo Fisher, Waltham, MA, USA), and QuantStudio 6 or 7 FLEX Real-Time PCR instruments (Applied Biosystems, Foster City, CA, USA). Data were analyzed by QuantStudio^TM^ Real-Time PCR Software v1.3 (Applied Biosystems, Foster City, CA, USA).

DUX4-regulated gene expression levels were monitored by evaluating the expression levels of four well-established DUX4-regulated genes: *MBD3L2, ZSCAN4, LEUTX*, and *KHDC1L* [19, 31, 32]. *AHSA1* and *RPL27* were used as reference genes for normalization purposes. Expression levels of the four DUX4-regulated genes were evaluated individually for each selected gene, as well as in an integrated manner by calculating the FSHD composite score. Calculations for the FSHD composite were as follows:

1. ΔCt = (Av. Ct 4 DUX4-target genes) – (Av. Ct 2 HKGs)
2. ΔΔCt = ΔCt (DUX4 siRNA treated) – ΔCt (mock transfection)
3. FSHD Composite = 2^(-ΔΔCt)^ × 100 (%)

*ACTA1* gene expression levels were determined using *AHSA1* as a reference gene, and relative to the control treatment using the 2^(-ΔΔCt)^ method [33].

### RNA sequencing (RNA-seq) and data pre-processing

RNA sequencing libraries were prepared at Azenta US, Inc. using the NEBNext Ultra RNA Library Prep Kit for Illumina using the manufacturer’s instructions (NEB, Ipswich, MA, USA), including enrichment of poly(A) transcripts using Oligo(dT) beads. Samples were sequenced on the Illumina HiSeq instrument using a 2 × 150 bp paired-end configuration. After trimming, RNA-seq reads were aligned to the human reference genome GRCh38 [34] using the STAR aligner v2.5.3a [35].

### RNA-Seq differential gene expression analysis and seed-based off-target profile evaluation

Raw gene counts were used for quality control and differential expression analysis. Raw counts were normalized to the total number of reads by calculating log_2_CPM (counts per million), and lowly expressed genes (average log_2_CPM<−3) were eliminated before differential gene expression analysis. Differential gene expression analysis was performed using the Limma 3.40.6 software [36]. Samples from the three different FSHD patient-derived myotubes (MB02, MB05, MB06) were grouped, and the cell line was used as a covariate.

*DUX4* siRNA seeds were defined as the 6-mer, 7-mer (2 ways), or 8-mer sequences of the bases 1‒8 from the 5′ end of the guide strand. Based on the differential expression analysis comparing cells treated with *DUX4* siRNA sequences versus non-targeting control siRNA, the cumulative distribution of the fold-change was examined for the expressed genes with seed match hits (6-mer, 7-mer, and 8-mer) in their 3′ untranslated region (UTR) and for a control set of genes, with 6-mer matches in their 3′ UTR for 10 simulated siRNAs (denoted as the background set). The effect size between each seed match length’s fold-change distribution and the background distribution was determined by calculating the area under the curve. The associated *P* value was calculated using a Kolmogorov–Smirnov test.

### RNAseq pathway analysis

Gene set enrichment analysis (GSEA) [37] was performed to characterize the biological functions of the differentially expressed genes. We utilized an R package, fgsea (https://bioconductor.org/packages/release/bioc/html/fgsea.html), that pre-ranked the genes by their log_2_ fold-change (log_2_FC), then tested for their enrichment in those pre-defined gene sets as canonical pathways (BioCarta subset, KEGG_MEDICUS subset, and Reactome subset) collected in the Human Molecular Signatures Database (MSigDB, v7.5.1) [38]. Pathways with normalized enrichment scores (NES) with *P* < 0.05 were chosen for further analysis. Pathways shared by no less than two comparisons are shown in the plot.

### RNA-seq data deposition to the GEO public repository

The GEO accession number for the RNA-seq data is GSE291267.

### Immunofluorescent staining of myotubes

Myotubes were washed with PBS (14190–144; Thermo Scientific) and fixed in 4% paraformaldehyde (15 710; EMS) for 10 min at room temperature, followed by permeabilization and blocking for 1 h at room temperature using PBS containing 0.5% Triton X-100 (X100-100 mL; Sigma-Aldrich), 5% donkey serum (AB7475; Abcam) and 0.05% Tween 20 (BP337-500; Fisher Scientific). A primary antibody against myosin heavy chain (MAB4470; R&D Systems) was diluted 1:500 in PBS containing 0.1% Triton X-100, 5% donkey serum, 0.05% Tween 20 for 1 h at room temperature. A secondary antibody (Goat anti-Mouse Alexa Fluor™ Plus 488, A32723; Thermo Scientific) was diluted 1:2000 in PBS containing 0.1% Triton X-100, 5% donkey serum, 0.05% Tween 20 for 1 h at room temperature. Nuclei were counter-stained with 1 µg/mL in PBS Hoechst (33 342; Thermo Scientific) for 10 min. Myotubes were washed with PBS and imaged with Molecular Devices ImageXpress automated microscope (20× magnification).

### Animal treatment and tissue collection

All animal studies were conducted following protocols approved by the local Institutional Animal Care and Use Committee (IACUC), in accordance with the regulations outlined in the US Department of Agriculture (USDA) Animal Welfare Act as well as the “Guide for the Care and Use of Laboratory Animals” [39].

Mouse studies were conducted at The Jackson Laboratory. Male mice of the strain ACTA1-MCM;FLExDUX4 (Tg(ACTA1-cre/Esr1*)2Kesr/J, JAX Stock# 025 750 hemizygous crossed with B6 (Cg)-Gt(ROSA)26Sortm1.1(DUX4*)Plj/J JAX Stock# 028 710 heterozygous) were used in this study. One control group included the Tg(ACTA1-cre/Esr1*)2Kesr/J (JAX Stock# 025 750) (hemizygous) strain without the human *DUX4* gene. Mice were 5–7 weeks of age. Materials in the study were blinded for genotype and treatment group. Veterinary care was available throughout the course of the study, and animals were examined by the veterinary staff as warranted by clinical signs or other changes.

During the study, there were some animal exclusions. In the 0.5 mg/kg group on day 14, two mice were excluded due to an incorrect genotype. In the vehicle group on day 42, one mouse was excluded due to a treatment inconsistency. Additionally, the day 14 sample from the tibialis anterior (TA) 6 mg/kg group for tissue concentration was lost during the tissue processing procedure.

Pharmacokinetic (PK) and pharmacodynamic (PD) experiments in mice were performed as one cohort, aiming for five animals per group. Systemic administration experiments in mice were performed in three cohorts, aiming for nine mice per group. In this functional experiment, the groups of mice 3-5 were treated with tamoxifen (TMX) purchased from Sigma (T5648) and dissolved in 100% ethanol (Sigma, E7023) to 200 mg/mL at 55°C and then diluted to 20 mg/mL in corn oil and stored at −20°C until the day of dosing. Groups 1 and 2 were treated with a vehicle (1% ethanol in corn oil). Mice in both experiments were dosed once with either DUX4 AOC or with vehicle PBS via a single intravenous (IV) bolus injection in the tail vein. On the final day of the study, mice were euthanized by CO_2_ asphyxiation. Tissue necropsy samples for gene expression analysis were collected at the indicated time points into Lysing Matrix D tubes (MP Biomedicals, Santa Ana, CA, USA) and snap frozen on dry ice. Samples for RNA-induced silencing complex (RISC) loading analysis were placed into Covaris tissueTUBE cryo bags and snap frozen in liquid nitrogen. All samples were stored at −80°C.

The NHP study was conducted at Altasciences using naive male cynomolgus monkeys aged between 2 and 3 years (*Macaca fascicularis*, Cambodia, supplied by Worldwide Primates, Miami, FL, USA; and Orient BioResource Center, Alice, TX, USA). Animals were socially housed in a temperature- and humidity-controlled environment. An automatic lightning system provided a 12-h light/dark cycle except during designated procedures. Housing conditions were maintained except when animals were separated for study-specific procedures. Animals were acclimated to the laboratory procedures for at least 14 days before initiation of dosing. Animals were randomly assigned to groups based on established social units. AOC 1020 or vehicle (PBS) was administered by IV infusion over 60 min (±6 min) using a temporary catheter inserted into a peripheral vein connected to a primed infusion line. The dosing solution was delivered using an infusion pump. Dose administration was completed within 6 h of completion of the formulation preparation. A detailed clinical examination, with attention to the injection site, was performed prior to each dosing and then 24 h after each dose administration. Animals were placed in a procedural cage for the examination. For punch biopsy sample collection of skeletal muscles, animals were sedated with ketamine and dexmedetomidine, and two punches per tissue were collected. The location of punch biopsies was anatomically defined for each time point. For necropsies, animals were sedated, weighed, and euthanized by an overdose of euthanasia solution. Terminal tissues were collected within 1 h of euthanasia. Tissues were placed in tubes (Omni Hard Tissue Homogenizing Mix, 2 mL reinforced tubes, nuclease, and microbial DNA-free part# 19–628D), flash frozen in liquid nitrogen, and stored at −80°C.

### siRNA quantification in tissue samples

Stem-loop RT-qPCR (SL-RT-qPCR) assays have been described previously [40]. A specific SL-RT-qPCR assay was designed to quantify the guide strand of siDUX4.6. Tissue homogenates were diluted into tris-hydroxymethyl aminomethane ethylenediaminetetraacetic acid (TE) buffer with 0.1% Triton X-100. Standard curves were generated by spiking siRNA at eight concentrations into the appropriate matrix for comparison with the samples.

### NanoString gene expression analysis in murine muscle tissue

Tissue samples were homogenized in TRIzol Reagent (Life Technologies Cat #15 596 018) on FastPrep-24 5G (MP Biomedicals, LLC, Irvine, CA, USA). After homogenization, RNA was isolated using chloroform phase separation in Phasemaker™ tubes (Life Technologies Cat# A33248). The aqueous phase was then moved to the silica-based spin plate from the Direct-zol-96 RNA isolation kit (Zymo Research, Irvine, CA, USA) and processed according to the manufacturer’s instructions. Hybridization reaction, prepared with RNA, probe sets (Integrated DNA Technologies), and PlexSet™ Reagents (NanoString Technologies, Inc., Seattle, WA, USA), was incubated in a SimpliAmp Thermal Cycler (Applied Biosystems, Foster City, CA, USA) for 16 h. For DUX4-responsive genes, 100 ng RNA was used. For *DUX4* mRNA detection, 1 μg RNA was used. Samples were analyzed by the nCounter™ FLEX Analysis System (NanoString Technologies, Inc., Seattle, WA, USA). The 56 DUX4-responsive murine genes for Nanostring panel were selected based on RNA-seq data available [41] and met the following criteria: (i) were upregulated in moderate FSHD mouse model, (ii) had a robust expression levels in moderate FSHD mouse model (>35 TPM) and (iii) showed highest differential expression level between moderate FSHD mouse model and control mice (Log_2_FC>1.5) [41]. The raw Nanostring counts were normalized to internal positive controls, housekeeping genes, and calibrator samples. For *DUX4* expression analysis, the mean of the signal from 3 *DUX4*-specific probes was calculated.

### RT-qPCR gene expression analysis in murine muscle tissue

Tissue samples were homogenized in TRIzol Reagent (Life Technologies Cat #15 596 018) on FastPrep-24 5G (MP Biomedicals, LLC, Irvine, CA, USA). After homogenization, RNA was isolated using the Direct-zol-96 RNA isolation kit (Zymo Research, Irvine, CA, USA). Purified RNA was converted to cDNA using the High-Capacity cDNA Reverse Transcription Kit (Applied Biosystems, Foster City, CA, USA). cDNA was analyzed by qPCR using TaqMan Fast Universal Master Mix II (Thermo Fisher, Waltham, MA, USA), and TaqMan probes for the genes of interest (Supplementary Table S3) (Thermo Fisher, Waltham, MA, USA), using QuantStudio 6 or 7 FLEX Real-Time PCR instruments (Applied Biosystems, Foster City, CA, USA). Data were analyzed by QuantStudioTM Real-Time PCR Software v1.3 (Applied Biosystems, Foster City, CA, USA). Pro-fibrotic *Col3a1, Fn1*, and *Tgfb1* gene expression levels were determined using *Ppib* as a reference gene, and relative to the TMX-only treated ACTA1-MCM;FLExDUX4 group using the 2^(-ΔΔCt)^ method [33].

### Quantification of siRNA loaded into the RISC

Cryopulverized tissue was homogenized with T-PER lysis buffer (Thermo Fisher, Cat# 78 510) supplemented with protease inhibitors (Thermo Fisher, Cat # 78 429), heparin (2 mg/mL), and EDTA (5 mM). A total of 100 μL of the homogenate supernatant was incubated with Dynabeads M-280 Streptavidin (Life Technologies, Cat# 11206D) and biotinylated anti-Ago2 antibody (Wako, Cat# 018–22 021) overnight at 4°C. After incubation, beads were thoroughly washed using DynaMag-96 Side Magnet (Life Technologies, 12331D). Elution of Ago2-associated siRNAs was performed in 150 μL of TE buffer with 0.1% Triton X-100 and by incubating at 95°C for 10 min in SimpliAmp Thermal Cycler (Applied Biosystems, Foster City, CA, USA). After elution, siRNA and ubiquitously expressed miR-16 were quantified using the SL-RT-qPCR assay.

### Treadmill running

Mice were acclimated to the testing room for at least 60 min. AccuPacer 4-Channel Mouse Treadmill with Motorized Grade Adjust (Omnitech Electronics) was used. A single mouse was placed on each lane in the treadmill compartment at 0° incline and allowed to acclimate for a minimum of 5 min. The treadmill belt was then started at 5 m/min, whereupon the stimulus shocker was set to produce a continuous current of 1.0 mA, 50 V delivered at a frequency of 1.5 Hz. After 5 min at 5 m/min, the speed was increased by 5 m/min every 5 min until 25 m/min. Exhaustion was defined as 15 continuous seconds spent in the stimulus area. The procedure timed out at 30 min. The distance run before exhaustion was evaluated.

### *In vivo* force measurement

Aurora *In Vivo* Force Measurement System (Aurora Scientific) was used. Mice were anesthetized with isoflurane. The stabilized hind limb was positioned horizontally, perpendicular to a footplate holding the foot. This setup was used to measure the torque of the dorsiflexion of the foot when the TA muscle contracted and opposed the dorsiflexion, thereby measuring isometric force. The footplate subsequently imposed a plantar flexion of the foot while the TA was stimulated tetanically to measure force during eccentric contraction. Muscle contraction was elicited by transcutaneous electrical stimulation of the peroneal nerve. Torque values (Nm) were normalized by body weight (Nm/g). Normalized torque values were plotted against stimulation frequencies between 0 and 150 Hz to yield a torque–frequency curve for each mouse, for the isometric measurement.

### Compound muscle action potential (CMAP)

CMAP was measured on the hind limb muscles after stimulation of the sciatic nerve. Mice were anesthetized for up to 5 min with 2% to 3% isoflurane in O_2_, and body temperature was kept constant. The stimulating electrodes were placed on each side of the sciatic nerve at the proximal thigh, and stimulus intensity was increased until the CMAP was maximized. The amplitude of the CMAP and corresponding stimulation intensity were recorded. Motor responses were recorded from an intramuscular needle electrode in the TA muscle.

### Muscle fibrosis quantification

Skeletal muscle tissues were fixed in 10% neutral buffered formalin (NBF) and embedded in paraffin. Deparaffinized 5-µm sections were hydrated in distilled water and stained with Weigert’s iron hematoxylin and Sirius red. After mounting, the extent of fibrosis (Sirius red) was measured by semi-automated image analysis.

### AOC 1020 binding to TfR1 by enzyme-linked immunosorbent assay (ELISA)

Cynomolgus monkey transferrin receptor 1 (cTfR1, Sino Biological 90253-C07H), mouse transferrin receptor 1 (mTfR1, Sino Biological, 50741-M07H), and human transferrin receptor 1 (hTfR1, Sino Biological 11020-H07H) were each diluted in Dulbecco’s PBS (Gibco 10 010 023) to 1 ng/µL. Then 50 µL/well of each was added to individual half-well high-binding plates (Costar 3690) before overnight incubation at 4°C. Tris-buffered saline with 0.05% Tween 20 (TBST, Cell Signaling 9997S) was used in all ELISA studies for plate washing. Plates were washed 4 times between steps at 100 µL/well. Following the wash step, plates were blocked with Superblock™ (Thermo Fisher, 37 535) for 1 h at room temperature. Plates were washed again before the addition of samples diluted in Superblock. AOC 1020, AV01mAb, or anti-mouse TfR1 antibody (custom-made, Genscript PROBIO lot number U6212HI16006/202 303) was diluted in Superblock before addition to plates at 50 µL/well across a range of concentrations to establish a binding curve. Plates with samples were incubated for 1 h at room temperature, washed, and secondary anti-human antibody for AV01mAb and AOC 1020 (Peroxidase AffiniPure Donkey Anti-Human IgG, heavy and light chain specific, Jackson ImmunoResearch 709–035-149) or secondary anti-mouse antibody for anti-mouse TfR1 antibody (Peroxidase AffiniPure Donkey Anti-Mouse IgG, heavy and light chain specific, Jackson ImmunoResearch 715–035-151) for anti-mouse TfR1 antibody was added at 50 µL/well. Plates were again incubated for 1 h at room temperature before washing. 3,3′,5,5′-tetramethylbenzidine (TMB) substrate (1-Step™ Ultra TMB-ELISA [Thermo Fisher, 34 028]) was added at 50 µL/well, and the plates were incubated for 5 min at room temperature, followed by the addition of 25 µL/well of 2N sulfuric acid stop solution (R&D Systems DY994). Plates were read at 450 nm using a 570 nm reference wavelength. Wells containing capture and secondary antibody without the sample antibody were used for background subtraction. Corrected absorbance values were analyzed for binding using the non-linear fit “Specific binding with Hill Slope.”

### Luciferase-reporter assay in HEK293 cells

Dual DUX4-luciferase reporter constructs were generated by inserting the human *DUX4* sequence (NM_001306068.3) into the psiCHECK^TM^-2 vector (Promega) containing two different luciferase reporter proteins. The Renilla luciferase is the primary reporter for the human DUX4 sequence inserted at the 3′ end of the Renilla gene. The firefly luciferase is used as an intraplasmid transfection normalizer.

Three different dual DUX4-luciferase reporter constructs were generated, which contained either the annotated human *DUX4* sequence (human siDUX4.6 target site construct – Sequence: CGACGGAGACTCGTTTGGA), or a single nucleotide mutation in the siDUX4.6 target site to mimic the cynomolgus monkey predicted siDUX4.6 target site sequence (cynomolgus monkey siDUX4.6 target site construct – Sequence: CGAAGGAGACTCGTTTGGA), or the siDUX4.6 target site (19 nucleotides) replaced by a different sequence (mutated target site construct – Sequence: ACAACAGACTTTAATGTAA).

HEK293 cells (ATCC, #CRL-1573) were grown in recommended media (90% DMEM High Glucose, 10% FBS, penicillin, and streptomycin) and seeded in triplicate in 96-well tissue culture plates without antibiotics 24 h prior to transfection. On the day of transfection, siRNAs and dual DUX4-luciferase reporter construct (30 ng/well) were formulated with Lipofectamine 2000 (Life Technologies, Waltham, MA, USA) and Opti-MEM (Life Technologies, Waltham, MA, USA), and co-transfection was performed following the manufacturer’s instructions. Forty-eight hours after transfection, Firefly and Renilla luciferase activities were measured using the Dual-Glo^®^ Luciferase Assay System (Promega, Madison, WI, USA) according to the manufacturer’s instructions in an Infinite^®^ M200 Pro plate reader (Tecan, Männedorf, Switzerland). The relative Renilla/Firefly luciferase ratio was calculated by first subtracting the background from the luciferase activity measurements (no-reporter transfection controls used as background reference) and then generating the relative ratio to the no-siRNA transfection control.

### Data analysis and visualization

Data were analyzed and/or graphed using GraphPad Prism 9.2.0.332 software (San Diego, CA, USA).

## Results

### siDUX4.6 is a potent and specific DUX4-Targeting siRNA

The siDUX4.6 sequence was selected based on its ability to potently and specifically reduce *DUX4* mRNA levels in primary myotubes derived from patients with FSHD with varying disease severity (Supplementary Table S2). DUX4-regulated gene expression is a well-established molecular signature of FSHD skeletal muscle [13, 17–19]. Due to the sporadic and low-level expression of *DUX4* in FSHD skeletal muscle *in vivo* and in FSHD cell models *in vitro* (GSE291267 gene expression data reported here) [11, 20–22], activity of *DUX4* siRNAs was assessed by evaluating the composite expression of 4 well-established DUX4-regulated genes (*MBD3L2, ZSCAN4, LEUTX, KHDC1L*) [19, 31, 32]. Out of 70 *DUX4* siRNAs screened (Supplementary Table S1), the siDUX4.6 sequence was identified as a promising candidate based on its ability to robustly inhibit DUX4-regulated gene expression (Fig. 1B; Supplementary Fig. S1A-D, Supplementary Table S4), and its activity across a panel of FSHD myotubes derived from patients with FSHD1 and FSHD2 (Fig. 1C; Supplementary Fig. S2; Supplementary Table S5). Moreover, we have monitored the expression of *ACTA1* as a myogenic differentiation marker upon siRNA treatment and shown that siDUX4.6 does not interfere with the formation of myotubes (Supplementary Fig. S1E). Concentration-response curves in 2 FSHD patient-derived myotube lines demonstrated robust 95% or higher reduction of the FSHD composite gene expression, with an EC_50_ of 0.13 to 0.67 nM for siDUX4.6 sequence (Fig. 1D; Supplementary Fig. S3; Supplementary Table S6). We have also demonstrated that the individual 4 DUX4-regulated genes that comprise the FSHD composite score behave concordantly with the composite score (Supplementary Figs S1, S2, S3; Supplementary Tables S4, S5, S6). This observation confirms the suitability of the FSHD composite score as a measurement of DUX4 activity. Additionally, a luciferase-reporter approach further demonstrated the specificity of siDUX4.6 for *DUX4* mRNA (Supplementary Fig. S10). In this system, siDUX4.6 showed robust concentration-dependent activity towards both the human and the cynomolgus monkey *DUX4* target sites, with complete loss of activity upon mutation of the target site into an unrelated sequence (Supplementary Fig. S10). Collectively, these data demonstrate the target engagement and specificity of siDUX4.6 towards *DUX4* mRNA.

**Figure 2.**
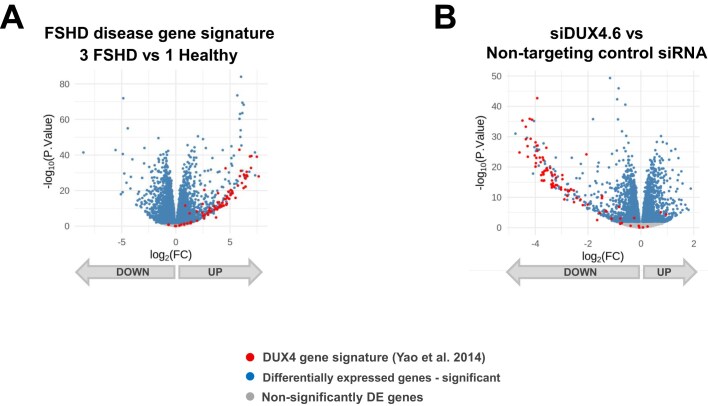
Transcriptome-wide effect of siDUX4.6 on DUX4-regulated genes in FSHD myotubes. Differential gene expression data are presented as volcano plots. One healthy (MB07) and three FSHD patient-derived primary myoblast cell lines (MB02, MB05, MB06) were treated with 10 nM siDUX4.6. Myotubes were collected in Trizol 3 days (MB02, MB05, MB07) or 4 days (MB06) after inducing differentiation, and gene expression was analyzed by RNA sequencing. The significance was established as FDR<0.05. (**A**) Average differential gene expression of three FSHD patient myotubes compared with one healthy donor myotube is plotted, all samples treated with non-targeting control siRNA. (**B**) Average differential gene expression of three FSHD patient myotubes treated with 10 nM siDUX4.6 versus non-targeting control siRNA is plotted. *n* = 4 for treatment in each individual cell line. Arrows at the bottom of the graphs indicate if genes were upregulated or downregulated. DE, differentially expressed; FC, fold-change; FDR, false discovery rate; FSHD, facioscapulohumeral muscular dystrophy; siRNA, small interfering ribonucleic acid.

Robust transcriptome-wide *in vitro* efficacy of siDUX4.6 was demonstrated in 3 FSHD patient-derived myotubes (Fig. 2). Using RNA sequencing, we performed global differential gene expression analysis in 3 FSHD myotubes following treatment with siDUX4.6 (Supplementary Table S7). A subset of 87 genes expressed in the FSHD myotubes had been previously identified as part of a DUX4-regulated gene signature characteristic of FSHD muscle (Fig. 2A) [19]. We observed that a large portion of these genes, 72 out of 87, were significantly upregulated in 3 FSHD patient-derived myotubes tested, compared with healthy myotubes (Fig. 2A). Of these 72 genes, 70 genes were significantly suppressed upon treatment with siDUX4.6 (false discovery rate, FDR < 0.05) in the 3 FSHD patient-derived myotubes (Fig. 2B). Additional analysis of the data revealed that 503 genes were upregulated in 3 FSHD myotube lines (log_2_FC≥1) with FDR < 0.05, and of these, 385 were suppressed with siDUX4.6 treatment, 198 significantly (FDR < 0.05). 279 genes were downregulated in 3 FSHD myotube lines (log_2_FC≤-1) with FDR<0.05, and of these, 191 were upregulated upon siDUX4.6 treatment, 11 significantly (FDR<0.05). To better understand the global effect of siDUX4.6 on myotubes, we performed a GSEA KEGG pathway analysis on the differential gene expression values of all genes regardless of statistical significance, to avoid any bias. Although the FSHD patient-derived myoblasts did not show the previously reported impaired differentiation phenotype [42, 43] with our culture and differentiation conditions and differentiated into fused multinucleated myotubes (Supplementary Fig. S11), we identified FSHD-specific pathways dysregulated in 3 FSHD myotubes evaluated by RNAseq. We report all pathways that are shared between at least two comparisons, in order to understand the (i) effect of siDUX4.6 on FSHD biology in cultured myotubes (these pathways are expected to be dysregulated in FSHD versus healthy and modulated in the opposite direction in FSHD cells treated with siDUX4.6), and (ii) general effect of siDUX4.6 on cells outside of FSHD biology, as such pathways would be similarly dysregulated in both FSHD and in healthy cells. We observe a siDUX4.6-mediated reversal of the majority of the pathways dysregulated in FSHD myotubes, with a minimal effect of siDUX4.6 on healthy myotubes (Supplementary Fig. S4). These data demonstrate a robust primary pharmacology of siDUX4.6 in FSHD patient-derived myotubes.

The microRNA-like off-target effects of an siRNA are mainly due to the guide strand’s seed region [44–46] (Supplementary Fig. S5A). We used RNAseq to evaluate the global effect of siDUX4.6 on the expression of genes containing a complementary siRNA seed sequence match within their 3′ UTR (unintended “bystander” transcripts) [47]. The differential gene expression profile largely overlapped with a background gene expression distribution in myotubes from one healthy donor and three FSHD donors (Supplementary Fig. S5B), demonstrating a negligible seed-based off-target profile of siDUX4.6.

### PK/PD profile of siDUX4.6 in the muscle of an FSHD mouse model

To assess *in vivo* target engagement of siDUX4.6, we utilized a transgenic mouse model of FSHD, ACTA1-MCM;FLExDUX4, with muscle-specific tunable expression of the human *DUX4* transgene [41, 48]. The ACTA1-MCM;FLExDUX4 mice express a TMX-inducible *DUX4* in skeletal muscle. However, even in the absence of TMX treatment, these mice have low background levels of *DUX4* expression that increases the expression of murine genes responsive to human DUX4 and produces a mild FSHD-like phenotype [41, 48]. We used a murine anti-TfR1 antibody to conjugate siDUX4.6 and generate a murine *DUX4* AOC since the antibody portion of AOC 1020 does not bind to mouse TfR1 (Supplementary Fig. S6).

**Figure 3.**
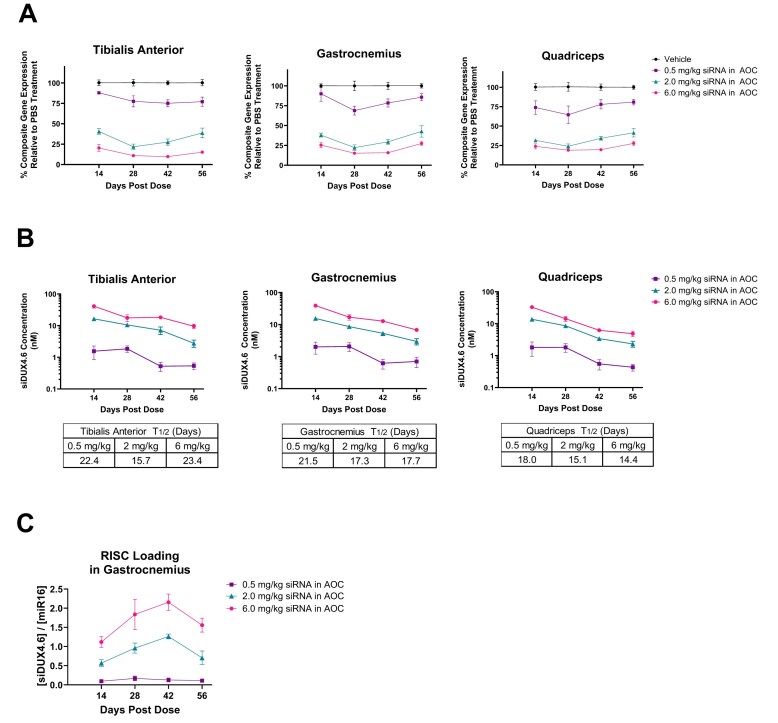
PK/PD properties of siDUX4.6 in the skeletal muscles in a mouse model of FSHD at varying doses. Murine *DUX4* AOC was administered intravenously in ACTA1-MCM;FLExDUX4 mice as a single dose. Skeletal muscle tissue analyses were performed at the indicated time points. (A) The level of target mRNA downregulation in skeletal muscle was determined by measuring 4 DUX4-regulated gene expression (*Wfdc3, Ilvbl, Slc15a2, Sord*), expressed as a composite gene expression relative to PBS vehicle-treated animals. (B) Muscle tissue concentration of siDUX4.6 was assessed by SL-RT-qPCR. siDUX4.6 tissue half-life was calculated by first applying a linear regression to the ln (natural log) tissue concentrations versus time profile and then using the resulting slope (λ = −slope) to calculate half-life with the formula: t_1/2_ = −0.693/λ. (C) RNA-induced silencing complex loading of siDUX4.6 in the gastrocnemius muscle was assessed as siDUX4.6 levels relative to miR-16 levels. *n* = 5 (except for sample exclusions detailed in the Materials and Methods); data expressed as mean ± SEM. AOC, antibody oligonucleotide conjugate; FSHD, facioscapulohumeral muscular dystrophy; PBS, phosphate-buffered saline; PD, pharmacodynamics; PK, pharmacokinetics; siRNA, small interfering ribonucleic acid; SL-RT-qPCR, stem loop reverse transcription quantitative polymerase chain reaction.

In a longitudinal study with the uninduced (i.e. no TMX treatment) mild FSHD mouse model, sustained dose-dependent siDUX4.6 activity was observed in skeletal muscle after a single IV dose of murine *DUX4* AOC, with approximately 75% inhibition of well-described DUX4-regulated murine genes [31, 48] at a 6 mg/kg dose (siDUX4.6 component) (Fig. 3A). Dose-dependent skeletal muscle exposure of siDUX4.6 was achieved after a single IV dose of murine *DUX4* AOC (Fig. 3B), with a tissue half-life of longer than 2 weeks (Fig. 3B). siRNA activity and its mechanism of action are dependent on its productive loading to the RISC [24, 49, 50]. Here, we assessed the loading of siDUX4.6 into the RISC in skeletal muscle by measuring the amount of siRNA detected in RISC isolated by Argonaute 2 (Ago2) immunoprecipitation. We observed dose-dependent RISC loading of siDUX4.6 in the gastrocnemius muscle. At the same time, siDUX4.6 RISC loading remained high in muscle for 8 weeks after a single IV administration of *DUX4* AOC at 2 and 6 mg/kg doses, which correlated with the observed robust and sustained *in vivo* activity (Fig. 3A,C). Overall, these data (Supplementary Table S8) demonstrate a productive delivery of siDUX4.6 into skeletal muscles of an FSHD mouse model, as well as a robust and durable primary pharmacology of siDUX4.6 *in vivo*.

### DUX4 AOC-mediated prevention of the FSHD phenotype in a mouse model

**Figure 4.**
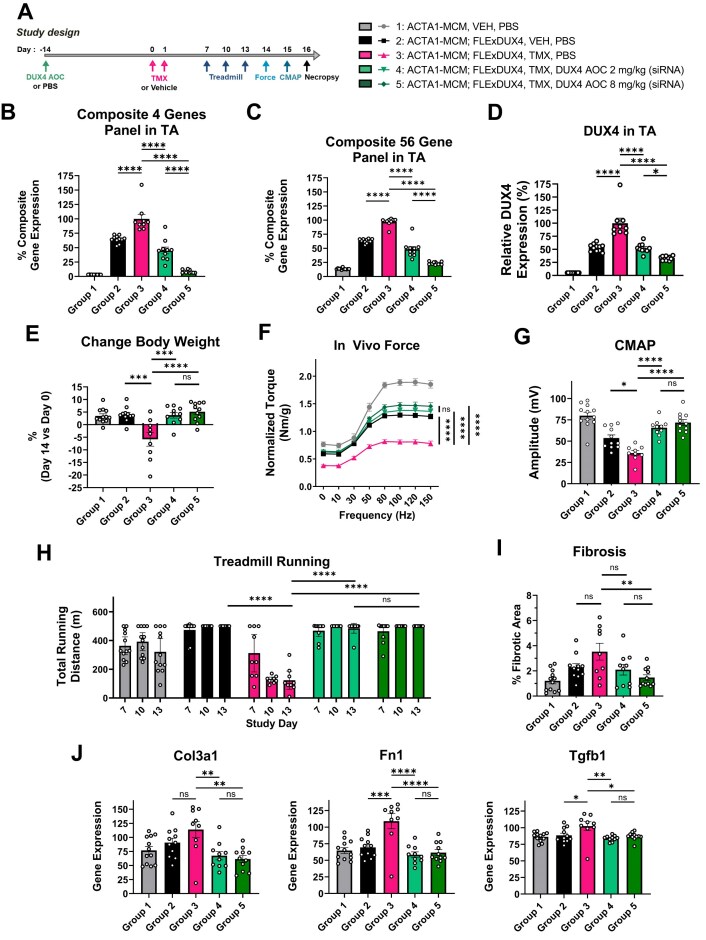
The effects of systemic administration of murine *DUX4* AOC on disease in a mouse model of FSHD. (**A**) Study design. Murine DUX4 AOC was administered once intravenously in ACTA1-MCM;FLExDUX4 mice at 2 or 8 mg/kg (siDUX4.6 within AOC) at day -14. TMX was administered on days 0 and 1 at a dose of 5 mg/kg. Control group 1: ACTA1-MCM mice that do not have the *DUX4* gene; control group 2: ACTA1-MCM;FLExDUX4 mice without TMX induction of *DUX4. n* = 12 for group 1; *n* = 11 for groups 2 and 5; *n* = 9 for group 3; *n *= 10 for group 4. (**B to D**) Gene expression was assessed 30 days post-DUX4 AOC administration. (**B**) Composite expression of DUX4-regulated genes in TA muscle was calculated as the geometric mean of 4 DUX4-regulated murine genes (*Wfdc3, Ilvbl, Slc15a2, Sord*). (**C**) Composite gene expression of the panel of 56 DUX4-responsive genes in TA was calculated as the geometric mean (**D**). *DUX4* expression was assessed in the TA muscle. (**E**) Total body weight was monitored at day 0 before TMX treatment and at day 14 of the study. (**F**) The in-life *in vivo* force measurements were performed on day 14. (**G**) In-life electromyography and CMAP were performed on day 15. (**H**) Treadmill running was performed on days 7, 10, and 13. (**I**) The extent of fibrosis developed in skeletal muscle was quantified in the quadriceps at day 16 of the study. (**J**) Expression of pro-fibrotic genes was assessed in TA muscle. Data in (**B-G**) and (**I-J**) are presented as mean ± SEM. Data in (**H**) are presented as means with 95% confidence intervals. For (**B-E** and **G, I, J**), statistical analysis was performed using one-way ANOVA with Tukey’s multiple comparison test. For (**F**) and (**H**), statistical analysis was performed using two-way ANOVA with Tukey’s multiple comparison test. In (**F**), statistical significance is shown for the 150 Hz point. The asterisks indicate statistical difference at adjusted P value: : * *P <* 0.05; ** *P <* 0.01; *** *P <* 0.001; **** *P <* 0.0001; ns, not significant. We are showing here statistical significance for selected group comparisons to avoid crowding of the figure and to highlight the DUX4 AOC treatment effect. AOC, antibody oligonucleotide conjugate; CMAP, compound muscle action potential; FSHD, facioscapulohumeral muscular dystrophy; PBS, phosphate-buffered saline; siRNA, small interfering ribonucleic acid; TA, tibialis anterior; TMX, tamoxifen; VEH, vehicle.

*DUX4* AOC suppresses TMX-induced elevated expression of *DUX4* and DUX4-regulated genes in the ACTA1-MCM;FLExDUX4 mice. As previously reported, TMX treatment resulted in an increased expression of DUX4-regulated genes across 3 different skeletal muscles in this FSHD mouse model, measured as either composite expression of 4 well-documented DUX4-regulated murine genes (Wfdc3, Sord, Ilvbl, Slc15a2) (Fig. 4B; Supplementary Fig. S7A,C) or as composite expression of 56 selected DUX4-driven genes previously identified as induced by DUX4 in this mouse model [41] (Fig. 4C; Supplementary Fig. S7B,D). Consistently, and despite the challenges due to its low expression levels, we were also able to show a direct increase in *DUX4* mRNA levels upon TMX treatment in this mouse model (Fig. 4D). We observed a dose-dependent suppression of TMX-induced *DUX4* and DUX4-driven gene expression across three different skeletal muscles 30 days after murine *DUX4* AOC treatment (Fig. 4B–D; Supplementary Fig. S7). Importantly, a more in depth gene expression data analysis in individual animals demonstrated a high correlation between expression of *DUX4* and four individual DUX4-driven murine genes (*R *= 0.87–0.92; Supplementary Fig. S8A), as well as a high correlation between *DUX4* and the composite score comprised of either 4 (*R *= 0.92; Supplementary Fig. S8B) or 56 DUX4-driven genes (*R*= 0.94; Supplementary Fig. S8C). Of note, we also observed a strong correlation of DUX4-driven gene expression levels among different muscles on the level of individual animals (Supplementary Fig. S8F).

Treatment with murine *DUX4* AOC prevented the exacerbated DUX4-induced disease phenotype. TMX treatment of ACTA1-MCM;FLExDUX4 mice further elevates the expression of *DUX4* and DUX4-regulated genes and exacerbates the FSHD-like disease phenotype [41, 48]. In this mouse model of FSHD, the development of DUX4-induced disease phenotypes (Fig. 4E–I) was similar to what has been previously reported [41]. The muscle function and overall decline in mice were assessed by measuring total body weight (Fig. 4E) as well as by three independent functional in-life assays: (i) treadmill running (Fig. 4H), (ii) *in vivo* muscle force measurement of the lower leg muscles (Fig. 4F), and (iii) electromyography measured as CMAP (Fig. 4G). Consistent with the suppression of *DUX4* and DUX4-regulated gene expression in this model (Fig. 4B–D; Supplementary Fig. S7), a single IV dose of murine *DUX4* AOC prevented body weight loss (Fig. 4E), muscle weakness, and manifestation of the disease phenotype at both tested doses (2 and 8 mg/kg siDUX4.6 component) (Fig. 4F–H). Next, we explored the relationship between DUX4-driven gene expression and functional outcomes in the FSHD mouse model at the level of individual animals. We observed a strong anti-correlation of the expression of the composite score of 4 DUX4-driven genes with muscle strength (*R *= −0.77; Supplementary Fig. S8D) as well as with the CMAP (*R *= -0.77; Supplementary Fig. S8E). To understand the effect of *DUX4* AOC on different muscles, we performed gene expression correlation analysis between muscles. We observe a strong correlation of DUX4-driven gene expression levels among different muscles on the level of individual animals (Supplementary Fig. S8F).

Finally, we also demonstrate the prevention of muscle fibrosis in this mouse model with the treatment of *DUX4* AOC (Fig. 4I and Supplementary Fig. S9). The ACTA1-MCM;FLExDUX4 mouse model of FSHD develops mild muscle fibrosis [41], and the DUX4-induced fibrosis was accompanied by an upregulation of pro-fibrotic genes (Tgfb1, Col3a1, and Fn1) in TA muscle (Fig. 4J). *DUX4* AOC efficiently suppressed the DUX4-mediated upregulation of these profibrotic genes in muscle at both 2 and 8 mg/kg doses (Fig. 4J). Overall, these data (Supplementary Table S9) demonstrate robust efficacy of siDUX4.6 in skeletal muscle *in vivo* in the mouse model of FSHD, measured by multiple functional endpoints.

### Pharmacokinetic Profile of AOC 1020 in NHPs

AOC 1020, where siDUX4.6 is conjugated to human monoclonal anti-TfR1 antibody AV01mAb, binds to both human and cynomolgus monkey TfR1 with comparable affinity (Supplementary Fig. S6). The conjugation of siDUX4.6 does not compromise AV01mAb binding to TfR1 (Supplementary Fig. S6).

**Figure 5.**
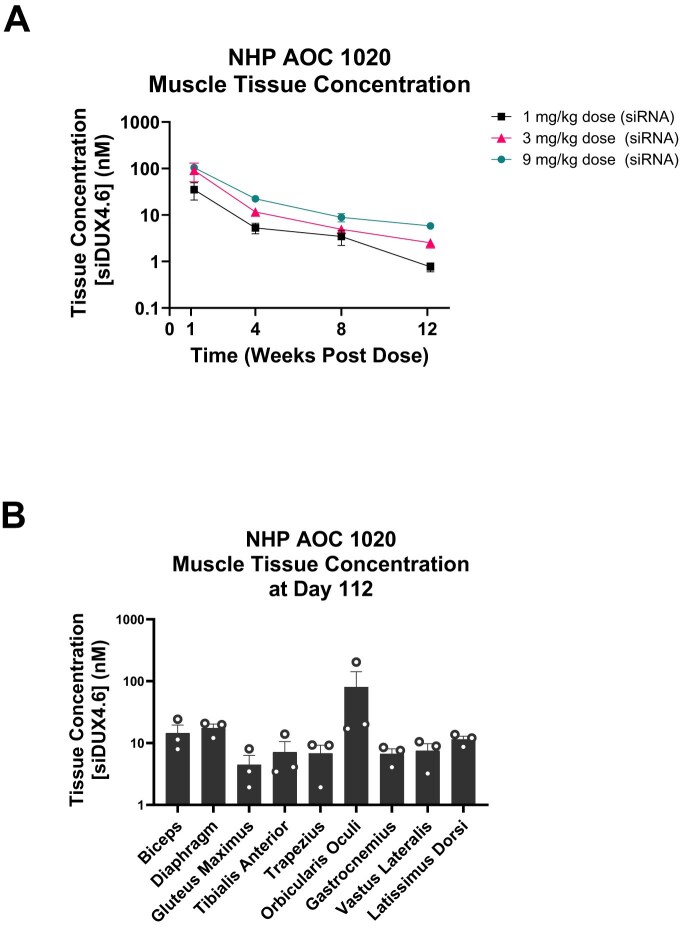
siDUX4.6 muscle tissue PK and exposure in non-human primates after systemic AOC 1020 administration. (**A**) The PK profile of AOC 1020 following a single dose for up to 12 weeks was evaluated in skeletal muscles, gastrocnemius, vastus lateralis, and latissimus dorsi of cynomolgus monkeys. AOC 1020 was administered by IV infusion at 1, 3, and 9 mg/kg (siDUX4.6 dose levels within AOC 1020) on day 1. Serial muscle punch biopsies were sampled at days 8, 28, and 56, and terminal necropsies were sampled at day 85. siRNA concentration in tissue was assessed by SL-RT-qPCR. *n* = 3 animals, 2 or 3 muscles analyzed for each animal (gastrocnemius and latissimus dorsi at day -9; gastrocnemius and vastus lateralis at day 8; latissimus dorsi and vastus lateralis at day 28; gastrocnemius and vastus lateralis at day 56, gastrocnemius, latissimus dorsi, and vastus lateralis at day 85); data are presented as mean ± SEM. (**B**) The distribution of siDUX4.6 at 16 weeks following repeat dosing of AOC 1020 was evaluated in the indicated muscles of Cynomolgus monkeys. AOC 1020 was administered three times by intravenous infusion at 3 mg/kg (reported as siDUX4.6 dose levels within AOC 1020) every 4 weeks (q4w), and terminal necropsies were collected at day 112 after the first dose. In tissue, siRNA concentration was assessed by SL-RT-qPCR assay. *n* = 3 animals; mean ± SEM. AOC, antibody oligonucleotide conjugate; NHP, non-human primate; PK, pharmacokinetic; siRNA, small interfering ribonucleic acid; SL-RT-qPCR, stem loop reverse transcription quantitative polymerase chain reaction.

Our *in vitro DUX4* target engagement data using the luciferase-reporter system (Supplementary Fig. S10) support that the cynomolgus monkey is a relevant NHP species for the pharmacology evaluation of siDUX4.6 and AOC 1020. As DUX4 is not expressed in the context of healthy muscle of NHPs, we focused on the evaluation of skeletal muscle tissue PK for AOC 1020 in cynomolgus monkeys. Following a single IV infusion of AOC 1020, increasing concentrations of siDUX4.6 were achieved in skeletal muscle in a dose-dependent manner over the dose range tested (1, 3, and 9 mg/kg, siDUX4.6 component) (Fig. 5A). The muscle tissue concentrations achieved 1 week post single AOC 1020 dose were between 10 and 100 nM. The half-life of siDUX4.6 in skeletal muscle tissue ranged between 15.7 and 19.3 days (Fig. 5A). These muscle tissue PK data in NHPs closely mirrored the muscle tissue PK data obtained in the mouse model of FSHD (Fig. 3B), indicating that siDUX4.6 is metabolically stable in muscle tissue across species.

For the distribution of siDUX4.6 across skeletal muscles, we focused on the muscles that are highly relevant to FSHD, such as facial muscles (orbicularis oculi), upper arm muscles (biceps brachii), shoulder girdle muscles (trapezius), back muscle (latissimus dorsi), pelvic girdle muscles (gluteus maximus), and leg muscles (gastrocnemius, vastus lateralis, and TA). After 8 weeks (day 112) following three doses of 3 mg/kg (siDUX4.6 component, every 4 weeks [q4w]), siDUX4.6 concentrations in skeletal muscles ranged from 4 to 100 nM (Fig. 5B; Supplementary Table S10).

## Discussion

### Rationale for development of AOC 1020

Identification of aberrant *DUX4* expression in skeletal muscle as the putative cause of muscle deterioration observed in patients with FSHD [8] triggered research efforts focused on developing therapeutic strategies directly targeting the *DUX4* mRNA transcript [51]. An oligonucleotide-based approach may provide advantages over other therapeutic modalities, including a straightforward oligonucleotide design and synthesis in addition to high selectivity toward the intended target [25]. Several oligonucleotide-based approaches targeting *DUX4* mRNA have been explored preclinically to date. In this preclinical setting, some studies demonstrated *DUX4* target engagement in the muscle of a mouse model of FSHD with an oligonucleotide approach [52, 53], while only a few demonstrated functional improvement [54–57]. In this report, we extend the previous *in vitro* and murine data by using a delivery system composed of a mAb targeting TfR1 conjugated to the siDUX4.6 therapeutic siRNA. We have recently demonstrated delivery of the siRNA therapeutic agent in NHPs and in patients [58]. It is challenging to bring promising preclinical oligonucleotide-based therapeutic advances to the clinic because of the combination of properties that such therapies need to exhibit. Some of the critical attributes of potentially successful medicines are potency, specificity, target tissue exposure, PK/PD profile, efficacy, and safety. The oligonucleotide field has witnessed advances as well as setbacks over the past decades, mainly due to an unfulfilled promise of delivery of therapeutic oligonucleotides of interest across a wider range of tissues beyond the liver and direct injections into the central nervous system [25].

We leveraged the strength of the AOC platform, which is translatable from rodents to NHPs [26], to develop AOC 1020 for the treatment of FSHD that fulfills all requirements for a potentially successful therapeutic. Importantly, AOC 1020 is the first oligonucleotide therapeutic agent for FSHD that has entered clinical evaluation (FORTITUDE, NCT05747924). AOC 1020 constitutes a novel therapeutic approach that combines the potency and specificity of an siRNA targeted directly toward *DUX4* mRNA with efficient anti-TfR1 antibody-mediated delivery to muscle. We have characterized the pharmacologic effects of AOC 1020 and its siRNA component, siDUX4.6, in several *in vitro* and *in vivo DUX4*-expressing preclinical models of FSHD.

### DUX4-regulated genes as surrogate biomarkers of DUX4 activity

The biology of DUX4 needs to be considered to successfully develop an efficacious *DUX4*-targeting medicine, as reliable measurement of DUX4 levels is challenging. *DUX4* possesses several unique challenges for the preclinical pharmacological evaluation of *DUX4*-targeted therapeutics: (i) limited understanding of functional homology between human *DUX4* gene and murine *Dux* gene [31, 59–63]; (ii) *DUX4* expression is reported to play a role during human zygotic genome activation in early embryonic development, after which time it is largely silenced in most healthy tissues including muscle, but aberrantly expressed in FSHD [13, 17]; (iii) ectopic *DUX4* expression in cell and animal systems results in a toxic gain of function and related toxicity [10]; (iv) *DUX4* encodes a transcription factor that is expressed at extremely low levels in a sporadic manner in few myonuclei in FSHD muscles [11, 20–22], therefore requiring measurement of DUX4-regulated gene expression as a proxy in human *in vitro* systems [19] and in mouse models of FSHD [31, 48].

DUX4 is a transcription factor that activates a network of downstream genes [16, 18, 19, 23, 32]. *DUX4* expression happens in a sporadic burst-like fashion in the skeletal muscle of FSHD patients, and afterwards the *DUX4* gene is relatively quickly shut down [20, 64], posing a challenge to directly measure *DUX4* expression in FSHD skeletal muscle. In contrast, the expression of DUX4-activated genes persists for a longer time, and work from multiple laboratories has demonstrated that DUX4-regulated gene expression is a robust surrogate biomarker of DUX4 activity in FSHD muscle [19, 41, 65, 66]. We have demonstrated robust suppression of DUX4-regulated genes in a variety of FSHD patient-derived myotubes *in vitro* by siDUX4.6, as well as in a mouse model of FSHD upon treatment with *DUX4* AOC. The strong correlation between expression of *DUX4* and the panel of either 4 or 56 DUX4-regulated genes in the muscle of the FSHD mouse model observed here and their coordinated response to DUX4-targeted treatment with *DUX4* AOC further supports the use of DUX4-mediated gene expression as a sensitive surrogate marker of DUX4 activity in FSHD. This concept is strengthened by the additional data we report here, demonstrating that an interventional therapy with *DUX4* AOC targeted to *DUX4* results in decreased DUX4-driven gene expression and correlates well with an improvement in functional endpoints in the mouse model of FSHD. Indeed, DUX4-regulated genes have been proposed as potential biomarkers to monitor disease progression, target engagement, and treatment response in the clinic [16, 17, 66, 67]. Several DUX4-regulated genes that are upregulated in our FSHD myotubes *in vitro* are also altered in skeletal muscle biopsies from individuals with FSHD [16, 19, 23]. Of those, an identified core subset of 4 DUX4-regulated genes (*KHDC1L, LEUTX, PRAMEF2, TRIM43*) [19] were among the strongest suppressed genes by siDUX4.6 in FSHD patient-derived myotubes in our study (Supplementary Table S7). Importantly, one of these genes, *KHDC1L*, has been recently identified as a potential novel circulating marker of DUX4 activity in FSHD [68], further supporting the relevance and importance of DUX4-regulated genes as biomarkers of DUX4 activity in FSHD disease. Measuring such a circulating biomarker has the potential to provide a practical and minimally invasive way to monitor disease activity, track progression, and evaluate therapeutic response in patients with FSHD.

### Selection of siDUX4.6 based on potency and minimal off-target profile

We opted for a siRNA approach to silence *DUX4*, as siRNAs have advantages over other oligonucleotide modalities, mainly the efficiency of RISC-mediated enzymatic activity resulting in an overall superior potency of siRNAs and their specificity and safety [69, 70]. For the selection of a potent *DUX4*-targeted siRNA, we have used myoblasts derived from muscle biopsies of patients with FSHD, and *in vitro* culture conditions that facilitate their myogenic differentiation into myotubes, thereby promoting expression of *DUX4* and DUX4-regulated genes [30], making this cell model well-suited for *DUX4*-targeted siRNA screening. Several highly active *DUX4*-targeting siRNAs were identified in our initial screening. The siDUX4.6 sequence was ultimately selected based on two additional criteria: (i) its robust activity across a panel of myotubes derived from a variety of patients with FSHD, and (ii) its minimal seed-based off-target profile.

Activity in patient cells with a wide range of disease severity across both FSHD1 and FSHD2 types is of particular importance, as the *DUX4* sequence and the single-nucleotide polymorphisms in the *DUX4* gene remain poorly annotated. siDUX4.6 demonstrated robust activity and subnanomolar potency *in vitro*, reducing the expression of selected DUX4-regulated genes across all FSHD donors tested, who represent a broad spectrum of the FSHD patient population. Additionally, transcriptome-wide gene expression analysis further demonstrated the efficacy of siDUX4.6 in 3 tested FSHD myotubes, correcting a previously identified DUX4-regulated gene expression signature [19].

siRNA-mediated off-target transcript silencing is sequence-dependent and is a fundamental feature of RNAi-mediated silencing, where the sequence complementarity. Specificity of the siRNA is key in establishing both the mechanism of action (efficacy) and potential for off-target hybridization-dependent effect (safety). Here, we not only provide strong evidence of the relevance of *in vitro* human FSHD disease and mouse models of the potential for siDUX4.6 to suppress the effects of DUX4 protein expression in FSHD muscle, but we also demonstrate that siDUX4.6 exhibits a remarkably negligible off-target profile by examining global gene expression in cultured muscle cells derived from FSHD as well as healthy donors. Collectively, these data support the selection of siDUX4.6 as a development candidate and its further evaluation in non-clinical studies and clinical trials.

### *DUX4* AOC-mediated modulation of FSHD phenotype in a mouse model

Here, we recapitulate the disease-like phenotype that was previously reported upon TMX-mediated induction of *DUX4* in the ACTA1-MCM;FLExDUX4 transgenic mouse model of FSHD, such as muscle fibrosis and muscle weakness measured by treadmill assay [41, 48]. We have extended the functional evaluation in this mouse model to *in vivo* muscle force measurement and electromyography measured as CMAP. Both of these in-life assays captured impaired muscle function in the FSHD mouse model treated with TMX. *In vivo* force was measured by recording the force generated during eccentric contraction in the TA muscle stimulated tetanically in anesthetized mice. This assay is thus a direct and unbiased evaluation of the muscle force, independent of the mice’s behavioral components.

We demonstrate that the observed total body weight loss, elevated muscle fibrosis, overall fatigue measured by treadmill run, reduced muscle force, and altered Electromyography (EMG) were exacerbated in this FSHD mouse model upon TMX-induced *DUX4* expression, indicating that these endpoints were suitable to evaluate *DUX4* siRNA functional efficacy *in vivo*. Moreover, the above-mentioned functional endpoint measurements are all relevant to the FSHD pathology observed in the FSHD patient population. In FSHD, early myopathic changes, such as muscle fibrosis, as well as physical fatigue and musculoskeletal pain, may contribute to the presence of reduced specific muscle force. EMG studies in patients with FSHD showed myopathic changes that correlated with patient clinical characteristics [71]. Another study demonstrated a high sensitivity of multi-motor unit potential EMG analysis in FSHD [72].

A single systemic IV administration of murine *DUX4* AOC at 2 mg/kg (siDUX4.6 component) suppressed the expression of DUX4-regulated genes and prevented the development of the FSHD-like phenotype in the mouse model. This result was demonstrated by all assessed endpoints: body weight loss and all three in-life functional assays: treadmill run, CMAP, and *in vivo* force. Muscle fibrosis and expression of pro-fibrotic genes in muscle were also reduced in these mice by *DUX4* AOC treatment. Of note, while a clear dose-response was observed in terms of DUX4 target gene expression in the TMX-induced FSHD mouse model, the 2 mg/kg siRNA dose was sufficient to provide comparable functional efficacy to the 8 mg/kg dose. These observations demonstrate the benefit of *DUX4* AOC treatment in this mouse model of FSHD and suggest that even moderate levels of *DUX4* mRNA reduction may result in phenotypic improvement and clinical benefit. The functional endpoint data reported here provide mechanistic support for the concept of targeting *DUX4* with AOC 1020 in the clinic, and its potential to manage FSHD disease in human patients.

### Muscle tissue PK/PD profile of siDUX4.6 in mice and NHPs

A durable PD effect of oligonucleotide as a therapeutic agent, which allows for a sustained suppression of *DUX4* over a long period of time, is highly desirable to enable an infrequent dosing regimen. Here, a single systemic IV treatment of the mouse model of FSHD with the murine *DUX4* AOC produced a dose-dependent and durable reduction of DUX4-regulated gene expression in muscle tissues for at least 8 weeks. Durable activity of siRNA therapeutics is achieved via the siRNA guide strand loading into the RISC and subsequent robust RISC-mediated enzymatic activity [25]. We observed, as expected, that the *DUX4* target reduction correlated with the dose-dependent levels of siDUX4.6 loaded into the RISC in muscle tissue. The highest RISC loading of siDUX4.6 was observed between days 28 and 42 for the doses of 2 and 6 mg/kg, the same time points at which the maximum activity was observed in muscle. This observation supports the ability of siDUX4.6 siRNA to engage its target *DUX4* in a productive manner and reduce its expression in muscle via a durable RISC-mediated mechanism of action.

A favorable muscle PK profile of siDUX4.6 was achieved in both mice and NHPs, despite the antibody portion of the AOC molecule being species-specific. We have previously demonstrated that anti-TfR1 mAb-based AOCs are active in both mice and cynomolgus monkeys following a single dose, demonstrating the efficient delivery and pharmacological activity of an AOC with our platform in skeletal muscle and the heart across species [26]. In this study, the muscle tissue PK profiles of *DUX4*-targeted AOCs were evaluated in an FSHD mouse model with murine DUX4 AOC, as well as in NHPs (cynomolgus monkeys) with AOC 1020. In both species, we observed comparable dose-dependent muscle tissue exposure of siDUX4.6 with a muscle tissue half-life of more than 2 weeks. As a result, a high muscle exposure of siDUX4.6 over a long period of time supported robust and sustained activity in the FSHD mouse model. Importantly, in NHPs, a comparable exposure of siDUX4.6 was observed across the panel of muscles relevant to FSHD pathology, such as facial muscles, upper arm and shoulder muscles, pelvic girdle muscles, and leg muscles. These data across species are of particular importance, as they demonstrate a uniform and efficient delivery of siDUX4.6 to skeletal muscle via systemic delivery of AOC 1020 – a requirement for a drug to achieve efficacy in FSHD over an extended period.

### Clinical evaluation of AOC 1020

There are no approved therapies for the treatment of FSHD. A few therapeutic approaches have been or are currently being evaluated in clinical trials that assess the effects of treatments that do not directly target DUX4. Safety and efficacy of losmapimod, a p38 inhibitor, have been evaluated in a Phase 3 clinical trial (NCT05397470), but it missed its primary endpoints. Safety, tolerability, PK/PD, and efficacy of RO7204239, an anti-myostatin mAb, will be evaluated in a Phase 2 clinical trial in adult patients with FSHD (NCT05548556).

In contrast to the indirect approaches others have tried, the oligonucleotide component of AOC 1020, siDUX4.6, is designed to directly target *DUX4*. The siDUX4.6 is a highly potent and efficacious siRNA targeting *DUX4* across a panel of FSHD donors. In the context of AOC, siDUX4.6 demonstrates favorable skeletal muscle tissue exposure after a single systemic IV administration that translates well from mouse to NHPs. This favorable muscle PK profile results in robust and durable activity in the muscle of the FSHD mouse model that leads to a functional improvement of FSHD disease-relevant endpoints. We have thus generated a comprehensive preclinical pharmacology data package supporting AOC 1020 as a potential novel FSHD therapeutic agent. AOC 1020 is the first oligonucleotide-based therapeutic molecule being evaluated in clinical trials that directly targets the cause of FSHD disease, ectopic expression of *DUX4*, with others following (NCT06131983). AOC 1020 is currently being evaluated in the FORTITUDE Phase 1/2 (NCT05747924), the FORTITUDE-OLE Phase 2 (NCT06547216), and the FORTITUDE-3 (NCT07038200) clinical trials that include both male and female participants, and have demonstrated early promising signs of *DUX4* target engagement, efficacy, and safety in adults with FSHD [58].

## Supplementary Material

gkag301_Supplemental_Files

## Data Availability

All available data have been included in the manuscript. Additional data are available on request from Avidity Biosciences, Inc., San Diego, CA. Supplementary data are available at NAR online. The GEO accession number for the RNAseq data is GSE291267.

## Notes

Present address: Seawolf Therapeutics, Inc., 9880 Campus Point Drive, Suite 210, San Diego, CA 92121, USA
