# Supplementary material for: Development of a DUX4-targeting antibody oligonucleotide conjugate as a therapy for FSHD
Source: Nucleic Acids Res. 2026 Apr 17;54(7):gkag301. doi: 10.1093/nar/gkag301 (PMC13120848; doi:10.1093/nar/gkag301)
Supplement: gkag301_Supplemental_Files [file gkag301_supplemental_files.zip › Malecova et al Resubmission - Supplementary Figures.pdf]

Supplementary Material

Development of a *DUX4*-targeting antibody oligonucleotide conjugate as a therapy for FSHD

Barbora Malecova, David Sala, Garineh M. Melikian, Rachel Johns, Gulin Erdogan, Marc Hartmann, Maryam Jordan, J. Danny Arias, Arvind Bhattacharya, Qingying Meng, Oliver Dansereau, Samuel W. Beppler, Venkata Ramana Doppalapudi, Hanhua Huang, W. Michael Flanagan, Arthur A. Levin

Supplementary Figures

A

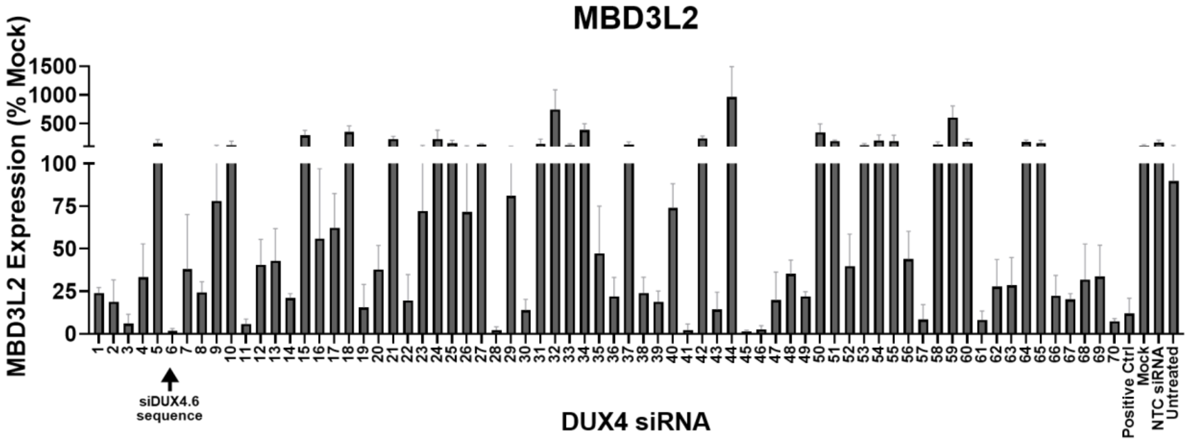

B

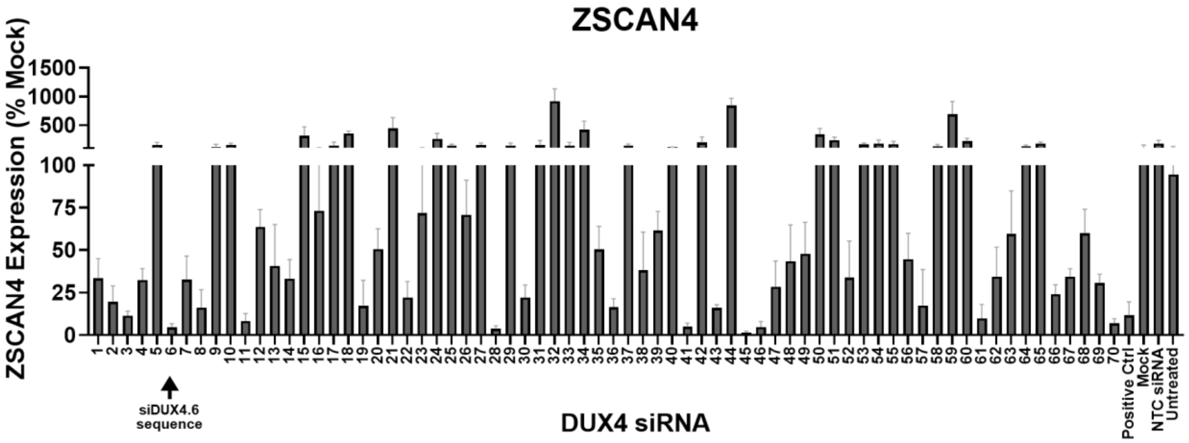

C

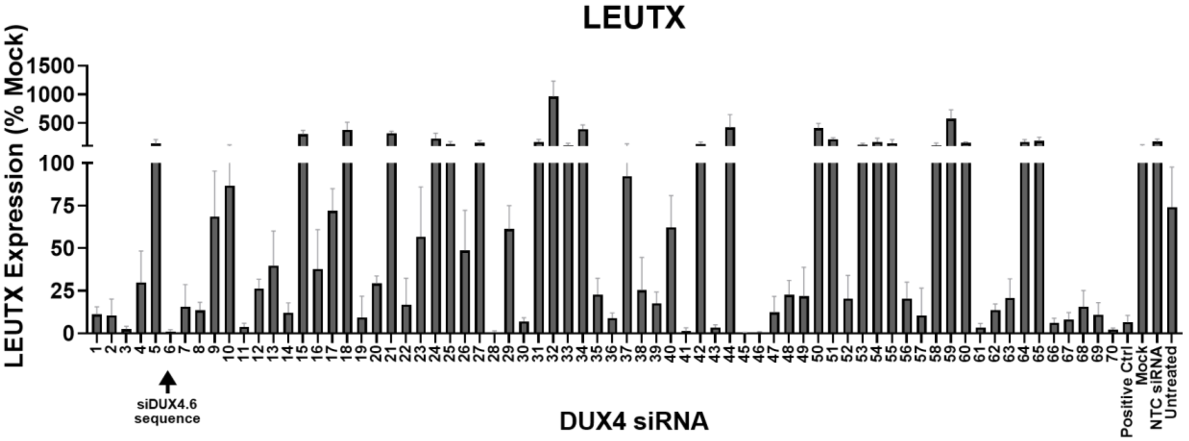

D

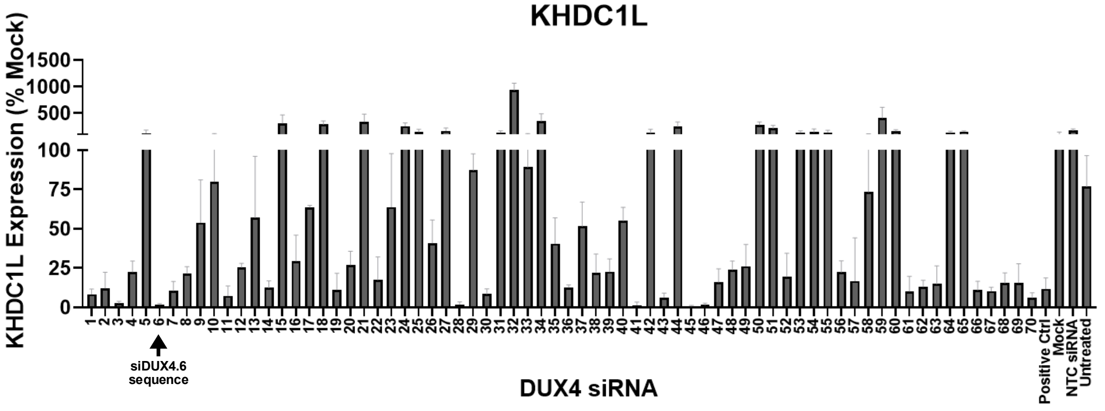

E

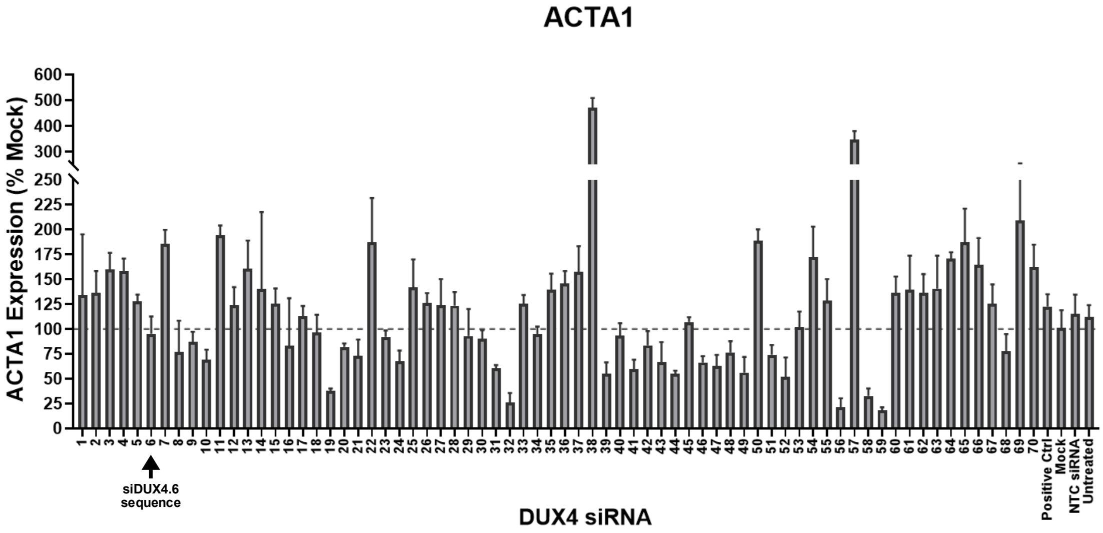

Supplementary Figure 1. *ACTA1* and individual DUX4-regulated gene expression for Figure 1A. Primary FSHD patient-derived myoblasts (MB06) were transfected with a library of *DUX4*-targeting siRNAs (10 nM) and induced to differentiate into myotubes to perform gene expression analysis. Gene expression levels of 4 selected DUX4-regulated genes (**A**) *MBD3L2,* (**B**) *ZSCAN4,* (**C**) *LEUTX*, and (**D**) *KHDC1L*, together with (**E**) *ACTA1* were evaluated. Data are represented as percentage of mock transfection control (mean ± SD, n = 4 for library siRNAs, n =8 for controls). CTRL, control; FSHD, facioscapulohumeral muscular dystrophy; NTC, non-targeting control; siRNA, small interfering ribonucleic acid.

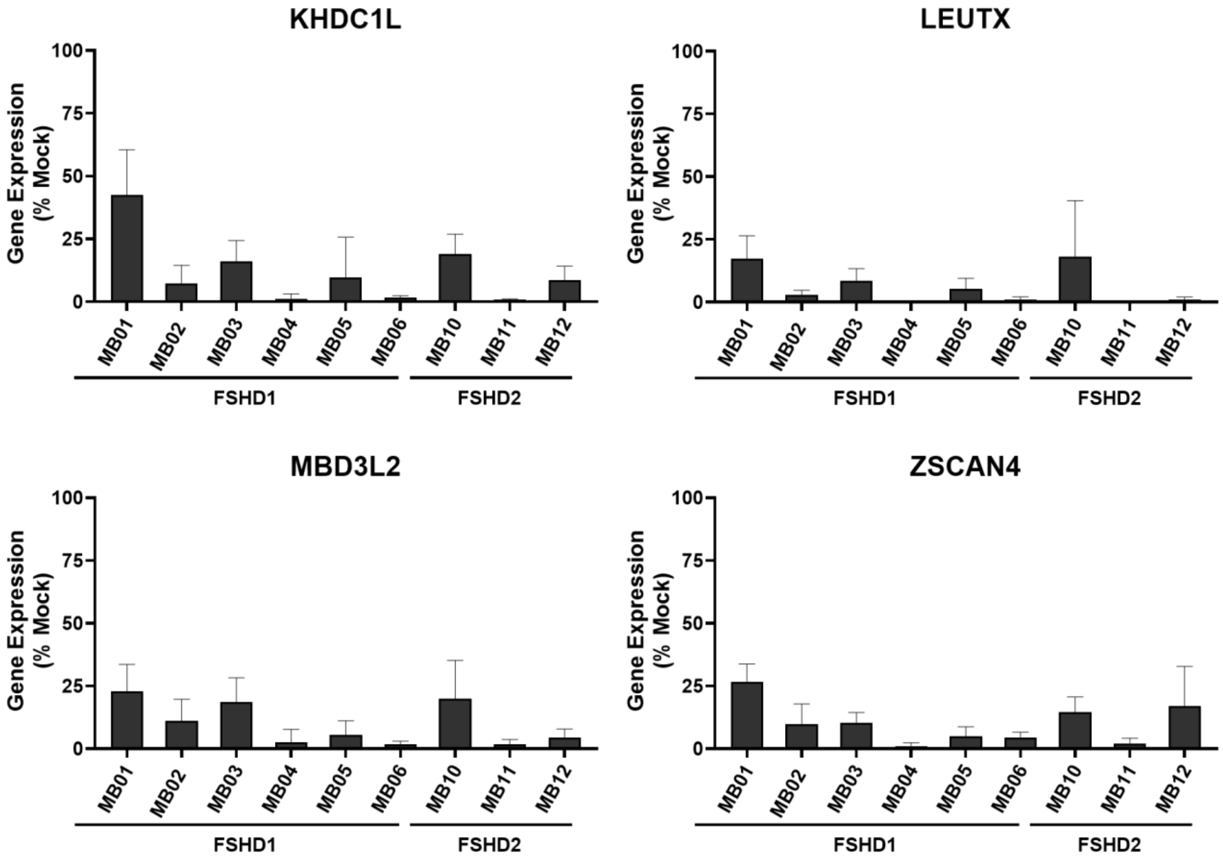

Supplementary Figure 2. Individual DUX4-regulated gene expression for Figure 1B. Activity of siDUX4.6 reducing the expression levels of 4 selected DUX4-regulated genes (*KHDC1L*, *LEUTX*, *MBD3L2*, *ZSCAN4*) was evaluated across 9 FSHD donor myotubes (MB06 data are the same as in Supplementary Figure 1; mean ± SD; *n* = 4 for siRNAs; *n* = 8 for mocks for MB03, MB04, and MB10; *n* = 4 for mocks for MB01, MB05, MB11 and MB12). FSHD, facioscapulohumeral muscular dystrophy; siRNA, small interfering ribonucleic acid.

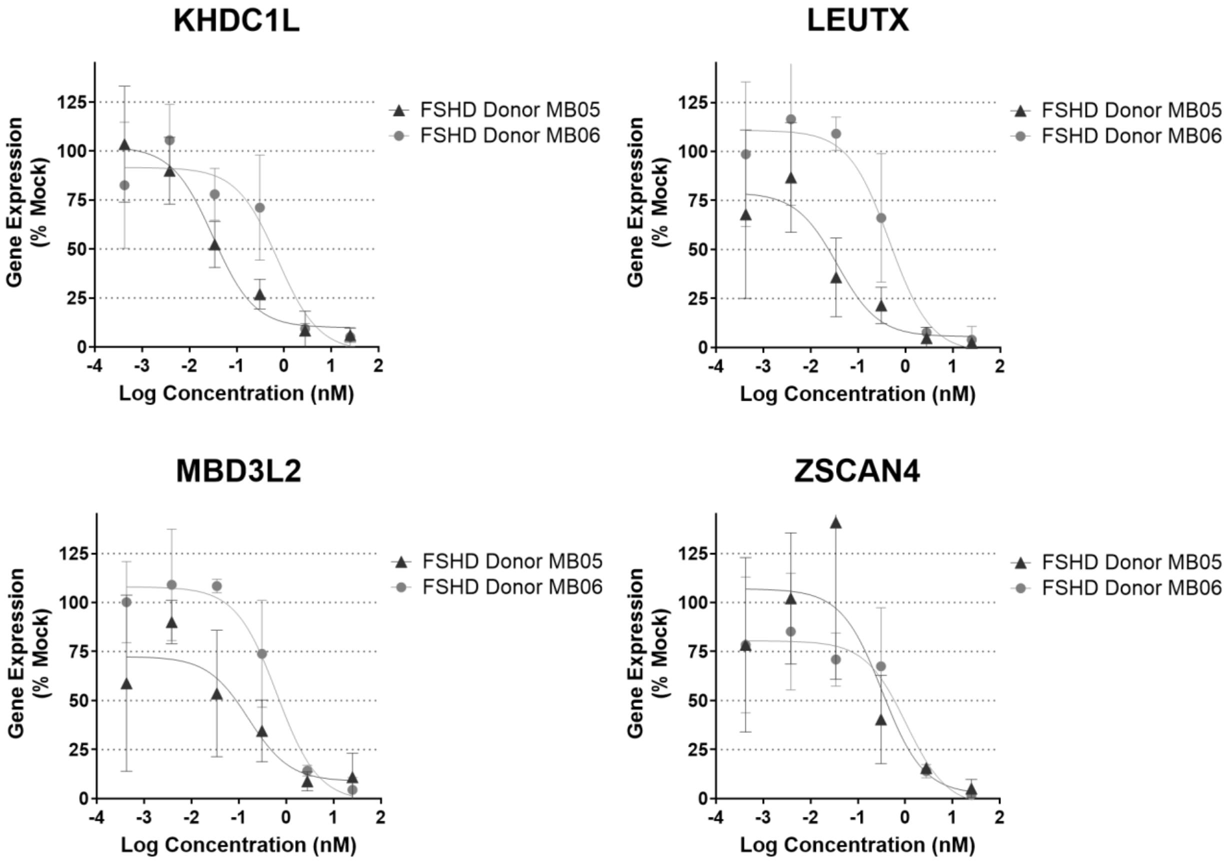

Supplementary Figure 3. Individual DUX4-regulated gene expression for Figure 1C. *In vitro* concentration-response in 2 FSHD1 patient-derived primary myotubes (MB05, MB06) was performed to evaluate siDUX4.6 potency. Graphs show the expression levels of 4 selected DUX4-regulated genes (*KHDC1L*, *LEUTX*, *MBD3L2*, *ZSCAN4*) as percentage of mock transfection control (mean ± SD, *n* = 4 for siRNA, *n* = 28 for mock). Log(inhibitor) versus response 3 parameters calculation was used to fit the concentration-response curves. FSHD, facioscapulohumeral muscular dystrophy; siRNA, small interfering ribonucleic acid.

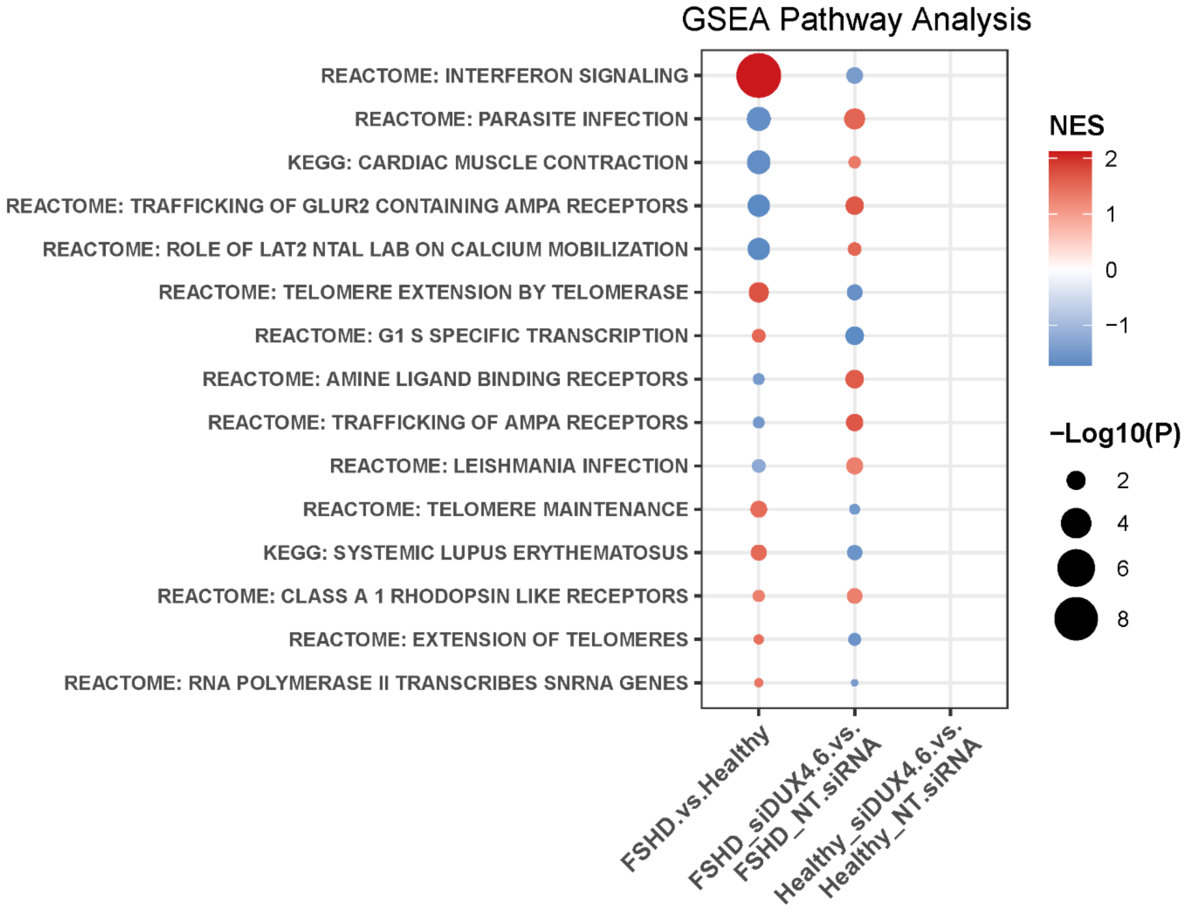

Supplementary Figure 4. GSEA was performed to characterize the biological functions of the FSHD-perturbed genes and the genes affected by siDUX4.6 treatment. The enriched pathways were arranged in the order of their NES and those with $P < 0.05$ were chosen for further analysis. Pathways shared by no less than 2 comparisons are shown in the plot. FSHD, facioscapulohumeral muscular dystrophy; GSEA, gene set enrichment analysis; NES, normalized enrichment scores; NT, non-targeting; siRNA, small interfering ribonucleic acid.

A

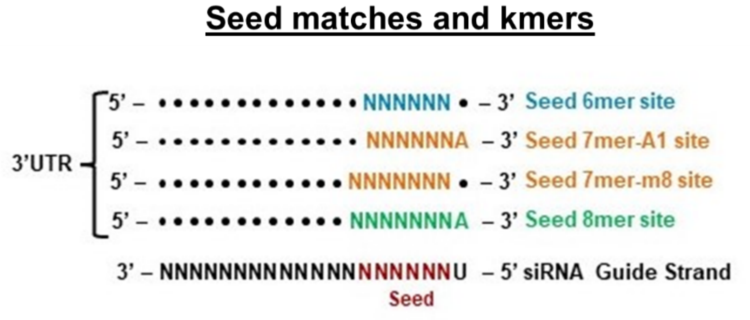
Seed matches and kmers
5' – •••••••••••••• NNNNNN • – 3' Seed 6mer site
5' – •••••••••••••• NNNNNNA – 3' Seed 7mer-A1 site
5' – ••••••••••••• NNNNNNN • – 3' Seed 7mer-m8 site
5' – ••••••••••••• NNNNNNNA – 3' Seed 8mer site
3'UTR
3' – NNNNNNNNNNNNNNNNNNNNU – 5' siRNA Guide Strand
Seed

B

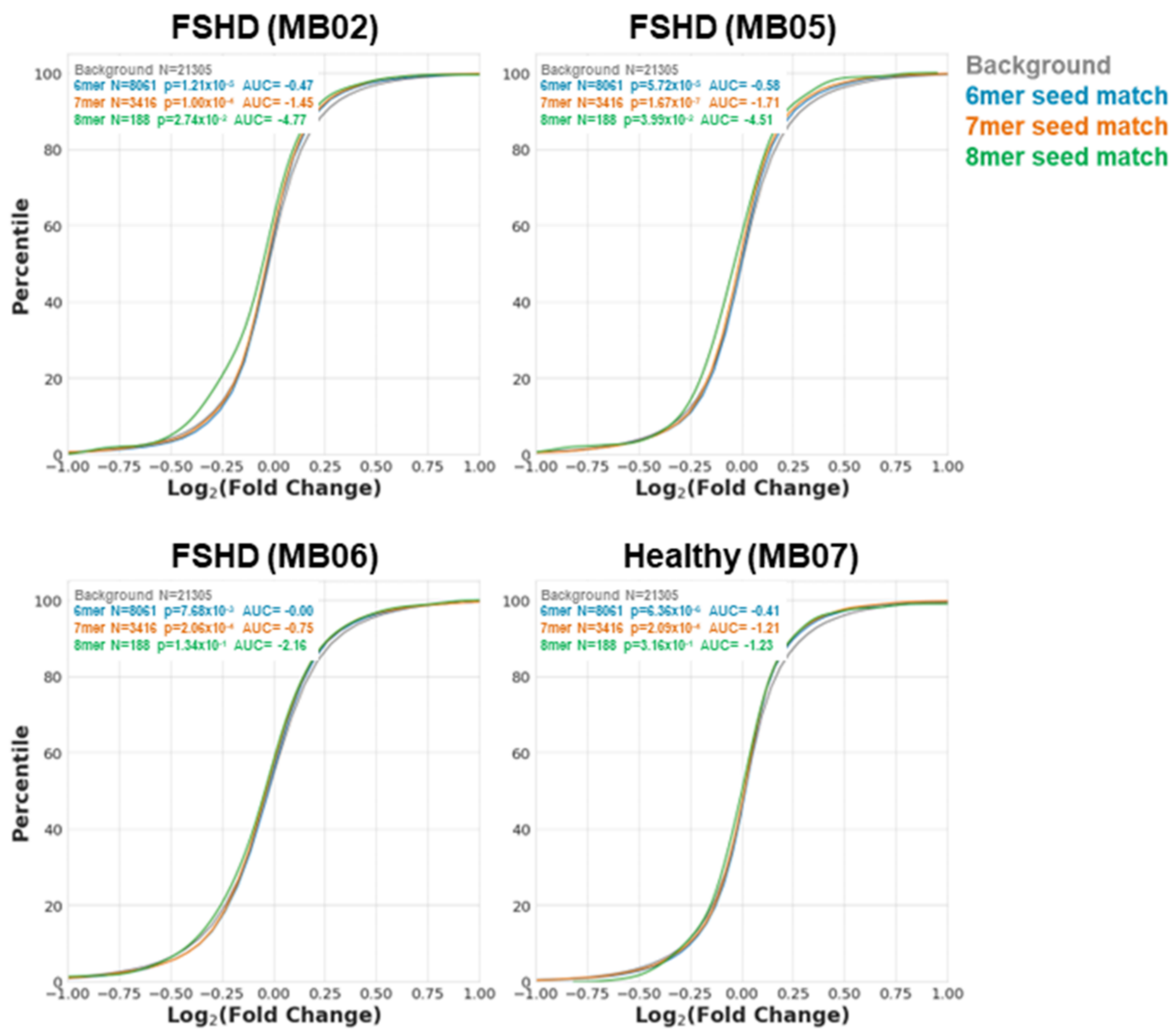
FSHD (MB02)
Background N=21305
6mer N=8061 p=1.21x10^{-5} AUC= -0.47
7mer N=3416 p=1.00x10^{-4} AUC= -1.45
8mer N=188 p=2.74x10^{-2} AUC= -4.77
FSHD (MB05)
Background N=21305
6mer N=8061 p=5.72x10^{-5} AUC= -0.58
7mer N=3416 p=1.67x10^{-7} AUC= -1.71
8mer N=188 p=3.99x10^{-2} AUC= -4.51
FSHD (MB06)
Background N=21305
6mer N=8061 p=7.68x10^{-3} AUC= -0.00
7mer N=3416 p=2.06x10^{-4} AUC= -0.75
8mer N=188 p=1.34x10^{-1} AUC= -2.16
Healthy (MB07)
Background N=21305
6mer N=8061 p=6.36x10^{-4} AUC= -0.41
7mer N=3416 p=2.09x10^{-4} AUC= -1.21
8mer N=188 p=3.16x10^{-1} AUC= -1.23
Percentile
Log$_2$(Fold Change)
Background
6mer seed match
7mer seed match
8mer seed match

Supplementary Figure 5. Negligible seed-mediated off-target profile of siDUX4.6 in human myotubes. (**A**) Scheme of siRNA seed region outlined as 6-mer, 7-mer, or 8-mer, used for the off-target assessment. (**B**) siDUX4.6 was analyzed for its off-target profile in cultured healthy and FSHD patient-derived myotubes by RNA sequencing. Differential gene expression distribution of the siDUX4.6 guide strand 3′ UTR-seed matched genes was assessed comparing the siDUX4.6 treated versus non-targeting control siRNA treated myotubes. Healthy (MB07) and FSHD1 (MB02, MB05, MB06) patient-derived primary myotubes were used (*n* = 4). AUC, area under the curve; FSHD, facioscapulohumeral muscular dystrophy; siRNA, small interfering ribonucleic acid; UTR, untranslated region.

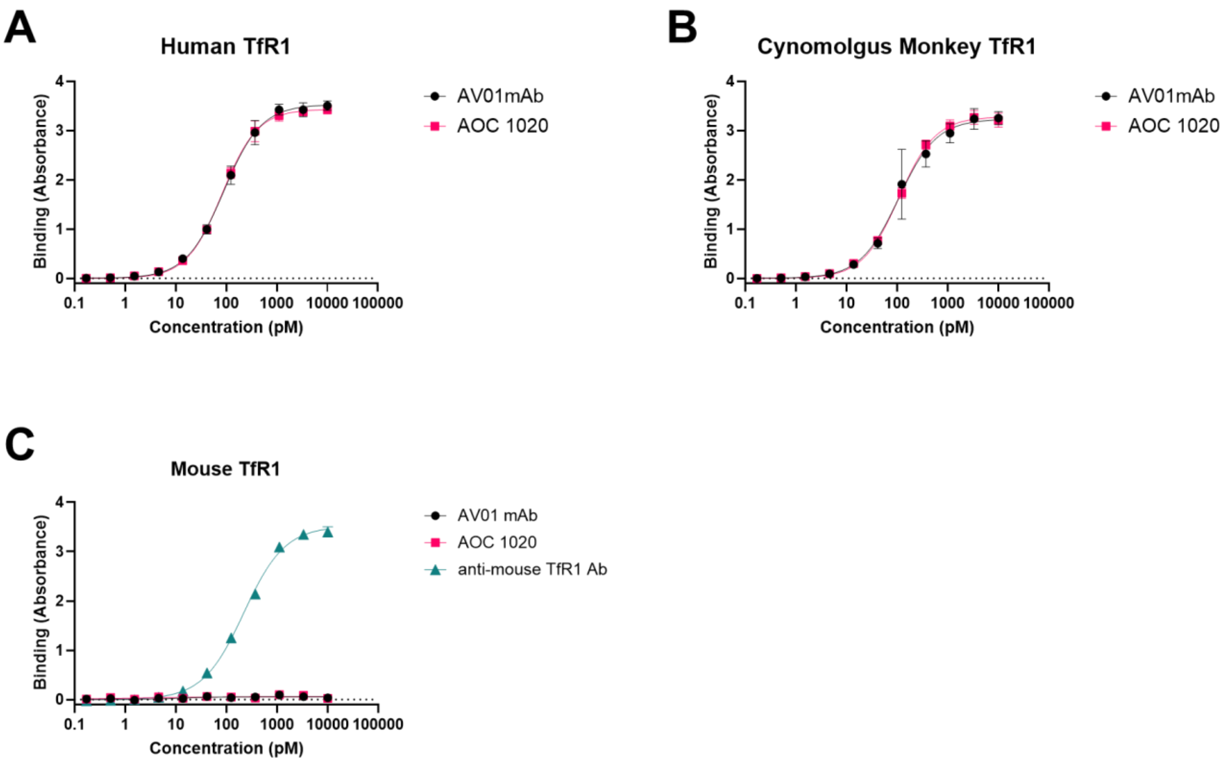

Supplementary Figure 6. AOC 1020 and AV01mAb bind to human and Cynomolgus monkey TfR1, but not mouse TfR1. AOC 1020 and AV01mAb binding affinity for (**A**) human, (**B**) cynomolgus monkey and (**C**) mouse TfR1 was measured by enzyme-linked immunosorbent assays. Mouse-specific anti-TfR1 antibody was added as a positive control for mouse TfR1. Data points represent mean ± SD (*n* = 4). Specific binding with Hill Slope was used to fit the binding curve using Prism GraphPad software and binding constant K_d was calculated based on the curve fit. The K_d of AV01mAb to human and Cynomolgus monkey TfR1 was 88.8 pM and 105.9 pM, respectively. The K_d of AOC 1020 to human and Cynomolgus monkey TfR1 was 82.7 pM and 108.6 pM, respectively. Ab, antibody; AOC, antibody oligonucleotide conjugate; mAb, monoclonal antibody.

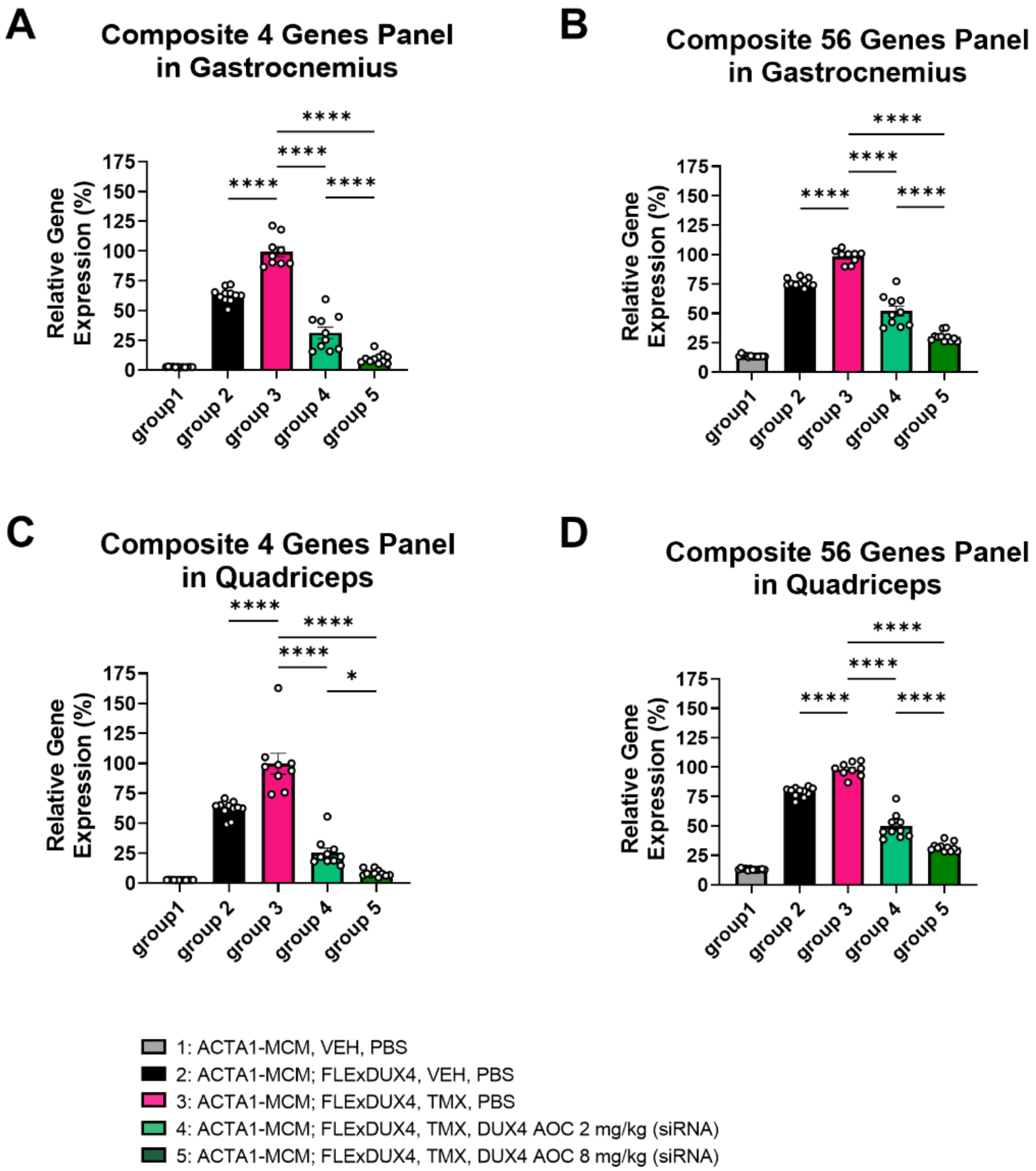

Supplementary Figure 7. DUX4-driven gene expression panel in gastrocnemius and quadriceps. (**A**) The composite expression of 4 DUX4-regulated murine genes (*Wfdc3, Ilvbl, Slc15a2, Sord*) and (**B**) the composite expression of the panel of 56 DUX4-responsive genes was assessed in gastrocnemius. (**C**) The composite expression of the 4 DUX4-regulated murine genes and (**D**) the composite expression of the panel of 56 DUX4-responsive genes were also assessed in quadriceps. N=12 for group 1; n=11 for groups 2 and 5; n=9 for group 3; n=10 for group 4. Statistical analysis was performed using one-way ANOVA with Tukey's multiple comparison test. The

asterisks indicate statistical difference at adjusted P value: * $P < 0.05$; **** $P < 0.0001$. AOC, antibody oligonucleotide conjugate; PBS, phosphate-buffered saline; siRNA, small interfering ribonucleic acid; TMX, tamoxifen; VEH, vehicle.

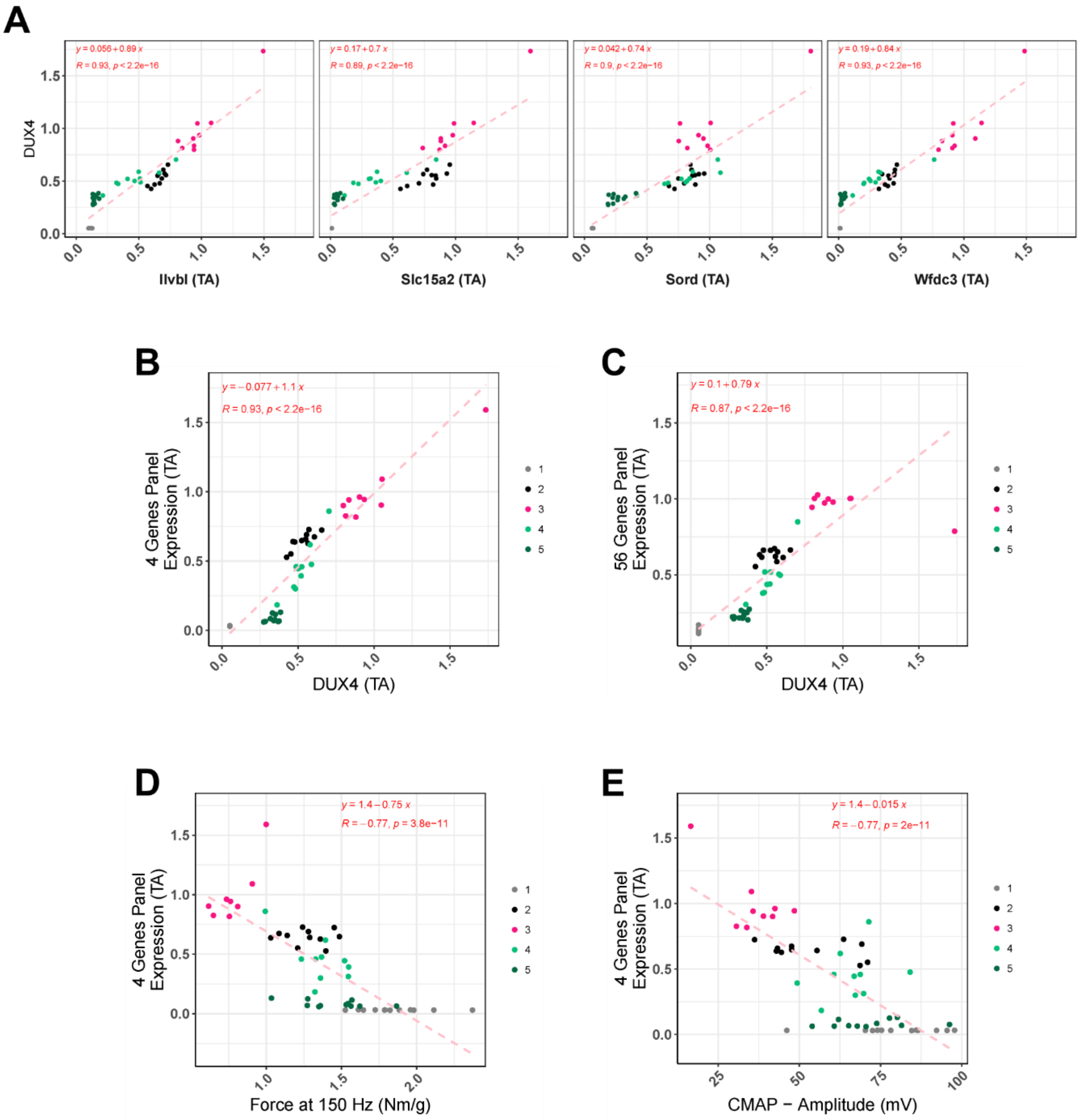

- 1: ACTA1-MCM, VEH, PBS
- 2: ACTA1-MCM; FLExDUX4, VEH, PBS
- 3: ACTA1-MCM; FLExDUX4, TMX, PBS
- 4: ACTA1-MCM; FLExDUX4, TMX, DUX4 AOC 2 mg/kg (siRNA)
- 5: ACTA1-MCM; FLExDUX4, TMX, DUX4 AOC 8 mg/kg (siRNA)

F

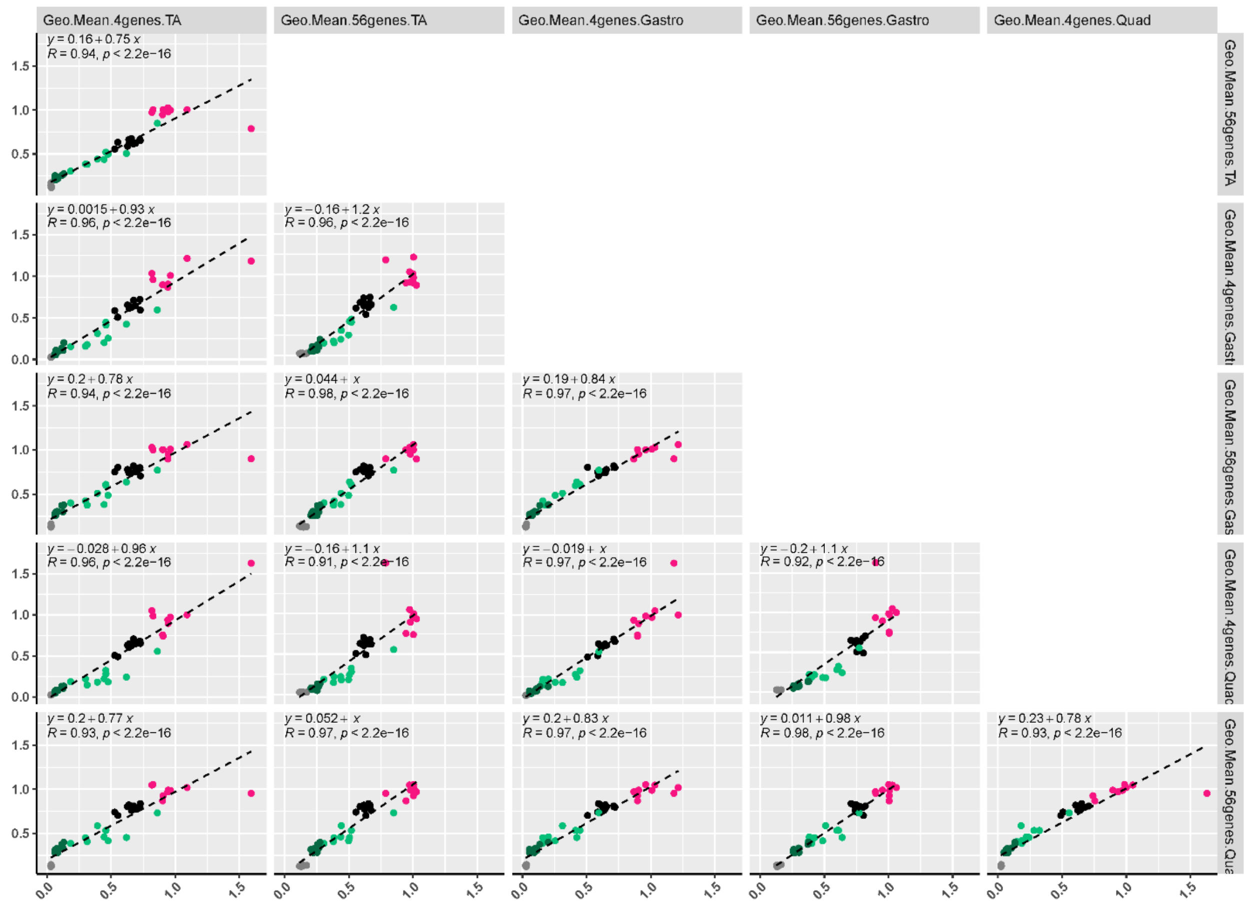

Supplementary Figure 8. Correlation of *DUX4* and DUX4-driven gene expression in TA muscle with force and CMAP in individual animals in the FSHD mouse model. (**A**) Correlation of *DUX4* expression with 4 individual DUX4-regulated murine genes *Wfdc3, Ilvbl, Slc15a2, Sord* in TA muscle. (**B**) Correlation of *DUX4* expression in TA with the composite expression of 4 DUX4-regulated murine genes (*Wfdc3, Ilvbl, Slc15a2, Sord*) in TA muscle. (**C**) Correlation of *DUX4* expression with the composite expression of 56 DUX4-responsive murine genes in TA muscle. (**D**) Correlation of the composite expression of 4 DUX4-regulated murine genes in TA muscle with normalized forced *in vivo*. (**E**) Correlation of the composite expression of 4 DUX4-regulated murine genes in TA muscle with CMAP *in vivo*. (**F**) Gene expression correlation analysis among individual muscles on the individual animal level. AOC, antibody oligonucleotide

conjugate; CMAP, compound muscle action potential; PBS, phosphate-buffered saline; siRNA, small interfering ribonucleic acid; TA, tibialis anterior; TMX, tamoxifen; VEH, vehicle.

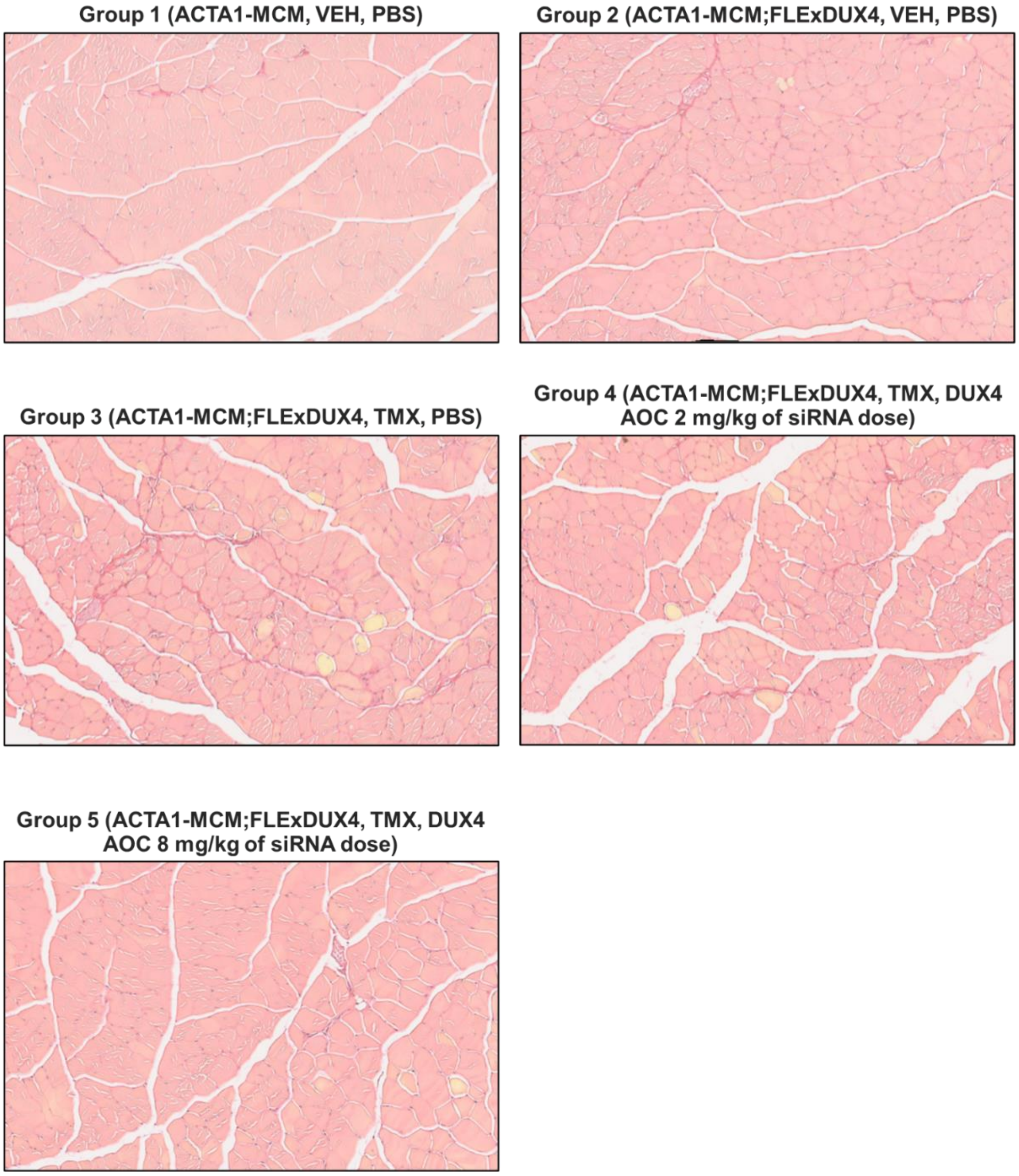

Supplementary Figure 9. Fibrosis evaluation in the skeletal muscle of the ACTA1-MCM;FLExDUX4 mice. Representative images of quadriceps muscle sections from ACTA1-MCM;FLExDUX4 mice with or without TMX and/or murine *DUX4* AOC treatments. Tissue sections were stained with sirius red. AOC, antibody oligonucleotide conjugate; PBS, phosphate-buffered saline; siRNA, small interfering ribonucleic acid; TMX, tamoxifen; VEH, vehicle.

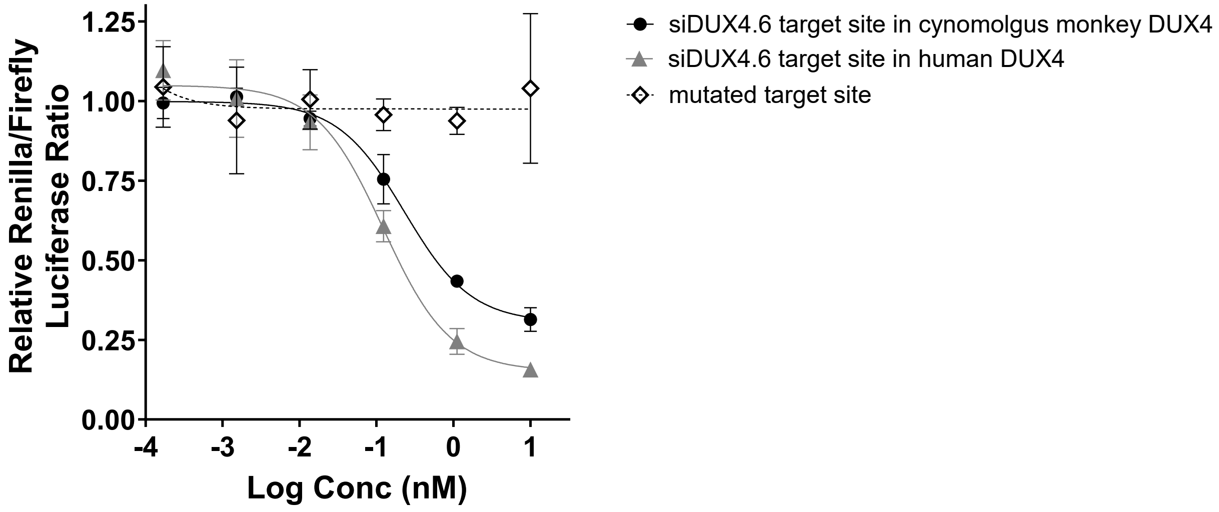

Supplementary Figure 10. siDUX4.6 is specific to the human and cynomolgus monkey *DUX4* target. HEK293 cells were co-transfected with a dual DUX4-luciferase reporter construct with the indicated final concentrations of siDUX4.6. Forty-eight hours after transfection, Firefly and Renilla luciferase activities were measured using the Dual-Glo® Luciferase Assay System (Promega, Madison, WI, USA). The Renilla luciferase activity was measured to monitor siDUX4.6 activity against three different dual DUX4-luciferase reporter constructs: (1) construct containing the human DUX4 target site; (2) construct containing the cynomolgus monkey predicted target site (1 mismatch for siDUX4.6); and (3) construct where the entire siDUX4.6 target site (19 nucleotides) was replaced by a different sequence (mutated target site). The Firefly luciferase activity was used as an intraplasmid transfection normalizer. Data in the graphs are represented as relative Renilla/Firefly luciferase ratio to no-siRNA transfection control (mean ± SD, n = 3). Log(inhibitor) versus response 3 parameters calculation was used to fit the concentration-response curves using GraphPad Prism 9.2.0.332 software (San Diego, CA, USA).

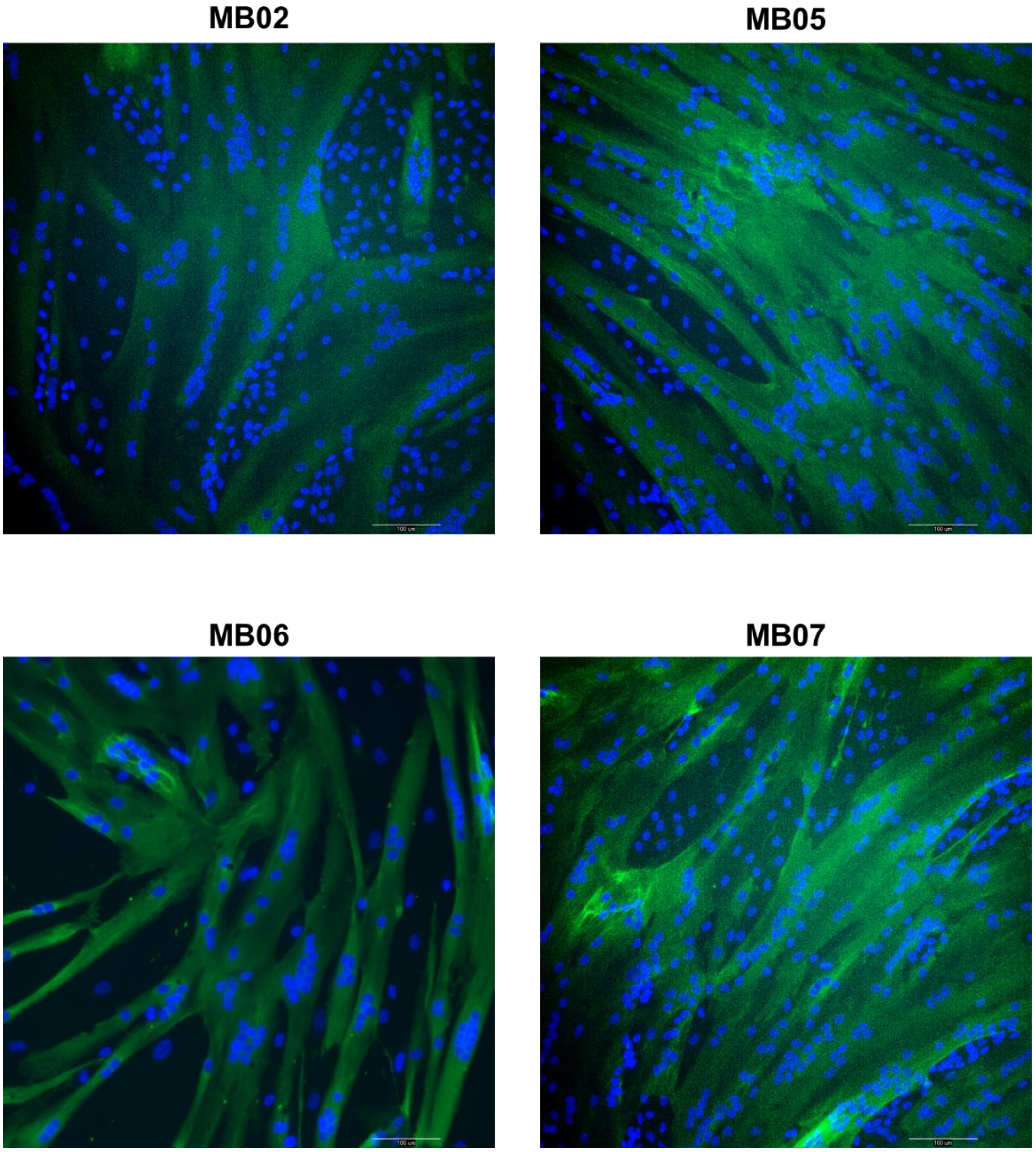

Supplementary Figure 11. Morphological evaluation of human myotubes. The propensity of FSHD (MB02, MB05, MB06) and healthy human (MB07) myoblasts to differentiate into myotubes was evaluated morphologically by microscopy. Myotubes were visualized by MHC staining (green). Nuclei were counter-stained by Hoechst (blue). Scale bars 100 µm.
